# Assessing the contribution of rare DNA states to cancer mutational signatures using sequence-specific conformational fingerprinting

**DOI:** 10.21203/rs.3.rs-8012102/v1

**Published:** 2025-11-19

**Authors:** Or Szekely, Yeongjoon Lee, Atul K. Rangadurai, Serafima Guseva, Joshua Cooksey, Edgar M. Faison, Nikita Zalenski, Qi Zhang, Zucai Suo, Hashim M. Al-Hashimi

**Affiliations:** 1 Department of Biochemistry and Biophysics, University of North Carolina at Chapel Hill, Chapel Hill, NC 27599, USA; 2 Department of Biochemistry and Molecular Biophysics, Columbia University, New York, NY 10032, USA; 3 Department of Biochemistry, Duke University School of Medicine, Durham, NC 27710, USA; 4 Department of Biomedical Sciences, Florida State University College of Medicine, Tallahassee, FL 32306, USA

**Keywords:** DNA dynamics, DNA damage, deprotonation, anion, mismatches, Hoogsteen, ^19^F NMR, *pK*_a_

## Abstract

Rare and short-lived DNA conformations are proposed to be key drivers of mutagenesis, yet assessing their contribution to mutational signatures found in human cancers remains challenging. Here, we developed an approach that quantifies the sequence-dependent propensity to form a rare DNA conformation and compares the resulting fingerprint against cancer mutational signatures. Using ^19^F NMR, we measured the propensity for the anionic Watson-Crick-like G•T^−^ conformation across all sixteen triplet sequence contexts and discovered a striking 50-fold variation driven by suboptimal interactions between anionic thymine and its 3’ neighbor. Comparing this fingerprint, and those of other rare DNA states, against the COSMIC database uncovered plausible links to mutational processes associated with exposure to damaging agents and therapies. Thus, integrating molecular biophysics with genomic epidemiology provides a powerful framework to explore how DNA’s dynamic properties shape genome stability and influence human disease.

## Introduction

**Figure 1.**
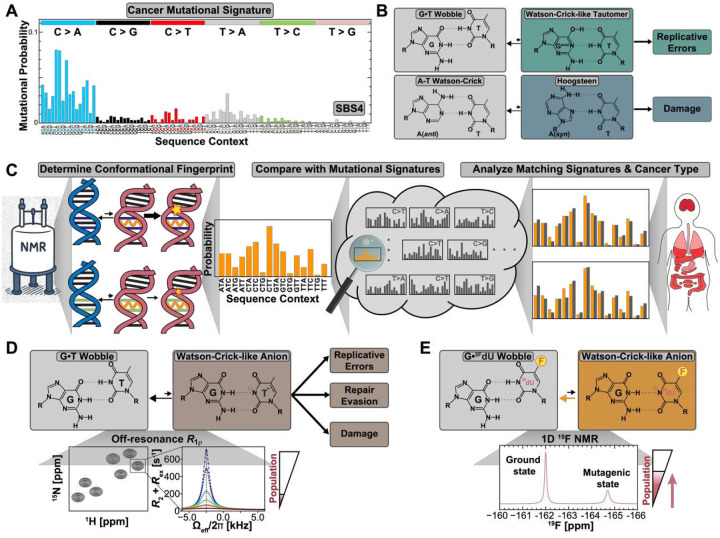
Assessing the contribution of rare DNA states to cancer mutational signatures using sequence-specific conformational fingerprinting. (A) Example of a single base substitution (SBS) mutational signature, showing the probabilities of six possible transition and transversion mutations (C>A, C>G, C>T, T>A, T>C and T>G) for each possible trinucleotide sequence context. Shown is the mutational signature SBS4 from the Catalogue of Somatic Mutations in Cancer (COSMIC) database of the Genome Reference Consortium Human genome build 37 (GRCh37)^36^, which is associated with tobacco smoking. (B) Rare DNA mutagenic states may play a role in different mutagenic processes. Examples of such rare states include the Watson-Crick-like tautomeric state of G•T mismatches, which can lead to replicative errors through misincorporation by DNA polymerases (top), and the transient A(syn)-T Hoogsteen conformation, which may lead to alkylation damage (bottom). (C) Propensities to form rare mutagenic states vary with sequence context yielding a unique conformational fingerprint. This sequence-dependence can impart itself on mutational processes acting on the mutagenic state. Comparison of these conformational fingerprints against the COSMIC database^36^ can be used to uncover plausible links to mutational signatures driven by the rare DNA state. The cartoon for cancer types (right) was adapted from Tomasetti et al^45^. (D) Measuring the propensities to form anionic WC-like G•T^−^ typically requires costly and time-consuming off-resonance relaxation dispersion NMR experiments on isotopically labeled DNA, which cannot easily scale across sequence space. (E) The T→^5F^dU substitution lowers the ${pK}_{\text{a}}$ of T(N3), bringing the WC-like G•T^−^ into direct detection via pH-dependent 1D ^19^F NMR, enabling the measurement of WC-like G•T^−^ across 16 sequence contexts.

Mutations in DNA are the driving force behind cancer. They arise from numerous processes, only some of which have been characterized at the molecular level^1–2^. Large-scale sequencing of human tumors^3–5^ has revealed that these processes leave behind distinctive *mutational signatures*, characteristic patterns of base substitutions that depend on both the mutation type and its surrounding DNA sequence^3, 5–8^ (Fig. 1A). Each signature is thought to reflect a specific mutational process. For example, lung cancers are enriched in C>A transversions because smoke-induced guanine adducts mispair with adenine during DNA replication^7, 9–10^ (Fig. 1A). Yet, the molecular origins of many mutational signatures remain elusive, and the role of DNA itself in shaping these patterns is not fully understood^11–15^. Elucidating how DNA actively shapes mutational landscapes is critical for elucidating cancer’s molecular origins and could ultimately inform strategies for early detection and therapeutic intervention.

One general mechanism by which DNA can influence mutagenic damage, damage repair, and replicative errors is by transiently adopting rare, short-lived conformational states^16–23^. For example, non-canonical mismatches can transiently adopt Watson-Crick-like (WC-like) conformations^17, 24–27^ through tautomerization or ionization of the bases and purine ring flips (Fig. 1B). By mimicking the Watson-Crick geometry, these rare states can evade DNA polymerase fidelity checkpoints and drive spontaneous misincorporation errors during replication (Fig. 1B)^17, 28–31^. Rare conformations can also expose otherwise protected sites to mutagenic damage^32^. For example, transient hairpin loops render apical loop cytosines susceptible to APOBEC3A deamination^33^ and A(*syn*)-T Hoogsteen base pairs (bps) expose adenine’s Watson-Crick face to alkylation damage (Fig. 1B)^34^.

Although a growing body of evidence supports the mutagenic potential of DNA transient states^16–17, 19–21, 28, 34^, assessing their contribution to mutational signatures observed in human cancers is difficult given their fleeting nature. Consequently, the extent to which these rare states shape mutational landscapes remains unknown even though they could underlie some of the cancer signatures currently lacking mechanistic explanation.

Here, we developed an approach to address this problem. Our central insight was that the probability of forming a rare DNA conformation also varies systematically with the identity of neighboring bps, generating a unique *conformational fingerprint (*Fig. 1C)^23, 35^. If mutational processes act through these rare conformations, their sequence-specific biases should propagate into characteristic mutational signatures observed across cancer genomes (Fig. 1C). Therefore, comparing experimentally determined conformational fingerprints against mutational signatures in the Catalogue of Somatic Mutations in Cancer (COSMIC) database^36^ could provide a means to assess the contribution of rare DNA states to mutagenesis *in vivo* (Fig. 1C).

This approach identifies only those mutational processes whose sequence dependence is primarily governed by a rare state. However, many mutational signatures arise from the combined influence of multiple biochemical steps, each with distinct sequence-specificities and possibly involving distinct DNA conformations. For example, mutagenic damage depends on the selective formation, repair, and replicative bypass of DNA lesions^32, 37^. The method further assumes that mutational processes are under thermodynamic control, determined by the equilibrium population of the rare DNA state rather than by its kinetic rate of interconversion^20, 38^. Finally, this correlative approach can only yield mechanistic hypotheses, and experimental verification will be needed to ultimately establish causation.

Even with these considerations, implementing this conformational fingerprinting strategy still poses major challenges, as it requires measuring the propensities of rare DNA states across all sixteen triplet sequence contexts. This is technically demanding, as these fleeting states typically populate at <1% and are detectable only through time-intensive NMR chemical exchange experiments^39–41^ that rely on costly isotopically labeled samples, limiting scalability across large sequence spaces (Fig. 1D). To overcome this challenge, we developed a sensitive and efficient ^19^F NMR strategy (Fig. 1E) that enabled comprehensive measurement of the propensity to form the mutagenic anionic WC-like G•T^−^ conformation across all sixteen triplet contexts.

The WC-like G•T^−^ state forms via deprotonation of the thymine base and its displacement toward the minor groove^24, 26, 28^ (Fig. 1D). By closely mimicking the WC geometry, this conformation can promote replicative errors both by facilitating misincorporation^17, 28^ and evading repair mechanisms^42–43^ (Fig. 1D). Replication errors are estimated to generate nearly two-thirds of all mutations in human cancers^44–45^, and G•T is the most frequently misincorporated mismatch^29, 46–49^. Left unrepaired, it produces T>C and C>T transition mutations, among the most prevalent substitutions in cancer^3, 7, 50^.

Beyond replicative errors, WC-like G•T^−^ could also contribute to mutagenic damage. In RNA, the related anionic inverted wobble G•U^−^ pair^51–55^ was recently shown to be highly reactive to dimethylsufate^56^ due to the increased nucleophilicity of uridine-N3. By analogy, the anionic G•T^−^ conformation in DNA could be susceptible to thymine-N3 alkylation damage, leading to formation of cytotoxic N3-methylthymidine (m^3^T) lesions^57–58^. Bypass of such lesions by translesion synthesis (TLS) polymerases often produces T>A, T>C, or T>G substitutions^58^. This anionic state is also noteworthy because intracellular pH is often elevated in cancers (>7.2^59^ and up to 7.6^60^) amplifying its mutagenic potential^28^.

Our ^19^F NMR-based strategy (Fig. 1E) builds on the pioneering work of Goodman *et al*^28, 61^ and is conceptually similar to the ^31^P phosphorothioate substitution approach by Bevilacqua *et al* for protonated A•C^62^. The approach replaces thymine with 5-fluoro-deoxyuridine (^5F^dU), which lowers the $pK_{a}$ of T(N3) by ~2.3 units^28, 61, 63–72^. This in turn boosts the anionic WC-like G•^5F^dU^−^ population ~100-fold^28^ enabling its direct detection and quantification under mildly basic conditions using 1D ^19^F NMR, leveraging the exquisite sensitivity of the ^19^F nucleus^61, 73–74^ (Fig. 1E). Assuming ^5F^dU uniformly promotes the anionic state across all sequence contexts^35^, the sequence-dependent preferences can be directly transferred to unmodified G•T^−^ (Fig. 1D). Comparing G•^5F^dU^−^ propensities with G•T^−^ measured using $R_{1\rho }$ in select sequence contexts, we demonstrate that this assumption holds to within an accuracy of ~0.3 kcal/mol.

The G•^5F^dU^−^ propensity exhibited dramatic 50-fold variation with sequence, driven by suboptimal interactions between the anionic thymine and its 3’ neighbor, with purines (G, A) causing greater destabilization than pyrimidines (C, T). Comparing this fingerprint, and those of other rare DNA states, against the COSMIC database uncovered plausible links to mutational processes associated with exposure to damaging agents and therapies. Thus, integrating molecular biophysics with genomic epidemiology provides a powerful strategy to explore how DNA’s dynamic properties shape genome stability and influence human disease.

### Design and NMR characterization of DNA library

**Figure 2.**
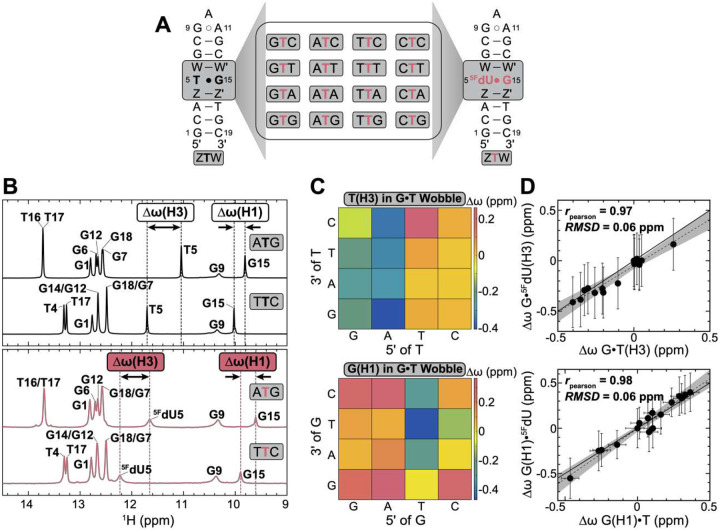
5F does not impact the sequence-dependent ensemble of the G•T wobble ground state. (A) DNA library comprising 2×16 triple sequence contexts with and without the 5F modification. Z-Z’ and W-W’ represent the four Watson-Crick (A-T, T-A, G-C, and C-G) neighbors. (B) Representative 1D ^1^H spectra showing sequence-dependent changes in chemical shifts in the absence (top) and presence (bottom) of the 5F modification. NMR spectra for all 16 sequence contexts are provided in Fig. S1. (C) Heatmap showing sequence-specific T(H3) and G(H1) imino ^1^H chemical shifts differences (Δω=ω(seq)-ω(ref); reference = CTA sequence context) in the G•T mismatch. Δω values are color-coded. Division into two groups by the 5’ neighbor is statistically significant based on Wilcoxon rank-sum test ($p=10^{-4}$ for T(H3), $p=10^{-2}$ for G(H1)). (D) Correlation plots comparing the sequence-specific chemical shift differences (Δω=ω(seq)-ω(ref); reference = CTA) measured for the sixteen DNA hairpins with and without the 5F modification. Error bars indicate the uncertainty in determining the peak position. The larger uncertainty for ^5F^dU(H3) is attributed to line-broadening due to the 5F modification (see panel (B) and Fig. S1B).

To implement the ^19^F-based NMR strategy, we adapted a DNA hairpin construct previously used in NMR studies of conformational exchange in the G•T mismatch^17^ (Fig. 2A). Starting from this construct, we generated a library encompassing all sixteen combinations of Watson-Crick bps flanking the central G•T mismatch, with either thymine or ^5F^dU at the mismatch position (Fig. 2A). Across all 2 × 16 hairpin constructs (Table S1), NMR chemical shifts (Fig. S1) and NOE-based distance connectivity (Fig. S2) measured at physiological pH revealed that the G•T/^5F^dU mismatch adopts a wobble ground-state conformation, with all other residues forming canonical Watson-Crick bps.

### 5F minimally impacts the sequence dependence of the G•T wobble conformation

Our approach assumes that although the 5F substitution increases the propensity to form the anionic WC-like conformation, this effect is systematic across sequence contexts, enabling transfer of sequence-specific propensities to unmodified G•T^−^. As an initial test, we used the thymine-H3 and guanine-H1 imino chemical shifts, which are sensitive to hydrogen bonding and ring currents from neighboring bases^75–77^ to examine whether 5F perturbs the sequence-dependent ensemble of the G•T wobble ground state.

Changing the triplet sequence context caused ~0.7 – 0.8 ppm chemical shift perturbations (CSPs) at T(H3) and G(H1) (Fig. 2B,C). The shifts were primarily dictated by the 5’ neighbor: T(H3) shifted upfield with 5’-purine and downfield with pyrimidine, whereas G(H1) shifted downfield with 5’-purine and upfield with pyrimidine (Fig. 2C). These contrasting sequence dependencies can be attributed to distinct ring current contributions at T(H3) and G(H1) from neighboring bps (Supporting Discussion 1 and Fig. S3A). Consistent with ring current effects, the CSPs arising from changing the identity of both WC neighbors (e.g. GTA→CTG) were to a good approximation (root-mean-square deviation, RMSD <0.16 ppm) given by the sum of the individual CSPs arising from changing the 5’ (e.g. GTA→CTA) and 3’-neighbor (e.g. GTA→GTG)^23^ (Fig. S3B).

The 5F modification induced the expected broadening of ^5F^dU(H3) and G(H1) imino resonances^72, 78–80^ across all sequence contexts (Fig. 2B, Fig. S1), reflecting faster solvent exchange kinetics from the ~2-unit $pK_{a}$ reduction^81^ and conformational exchange with the anionic WC-like state (see below). It also induced chemical shift perturbations, shifting ^5F^dU(H3) ~0.5 – 0.7 ppm downfield and G(H1) ~0.1 – 0.3 ppm upfield. However, these CSPs were largely systematic across the sequence contexts so that the sequence-specific H3 and H1 chemical shift differences relative to a reference sequence context (Δω=ω(seq)-ω(ref); reference = CTA) were in quantitative agreement with their unmodified counterparts, with RMSD of 0.06 ppm and $r_{\text{pearson}}\geq 0.97$ (Fig. 2D). The modification also did not impact (RMSD <0.14 ppm) the chemical shift additivity (Fig. S3C).

Taken together, these results indicate that the 5F modification does not significantly alter the sequence dependence of the G•T ground-state ensemble.

### Measuring sequence-specific propensities of G•^5F^dU^−^ using pH-dependent 1D ^19^F NMR

**Figure 3.**
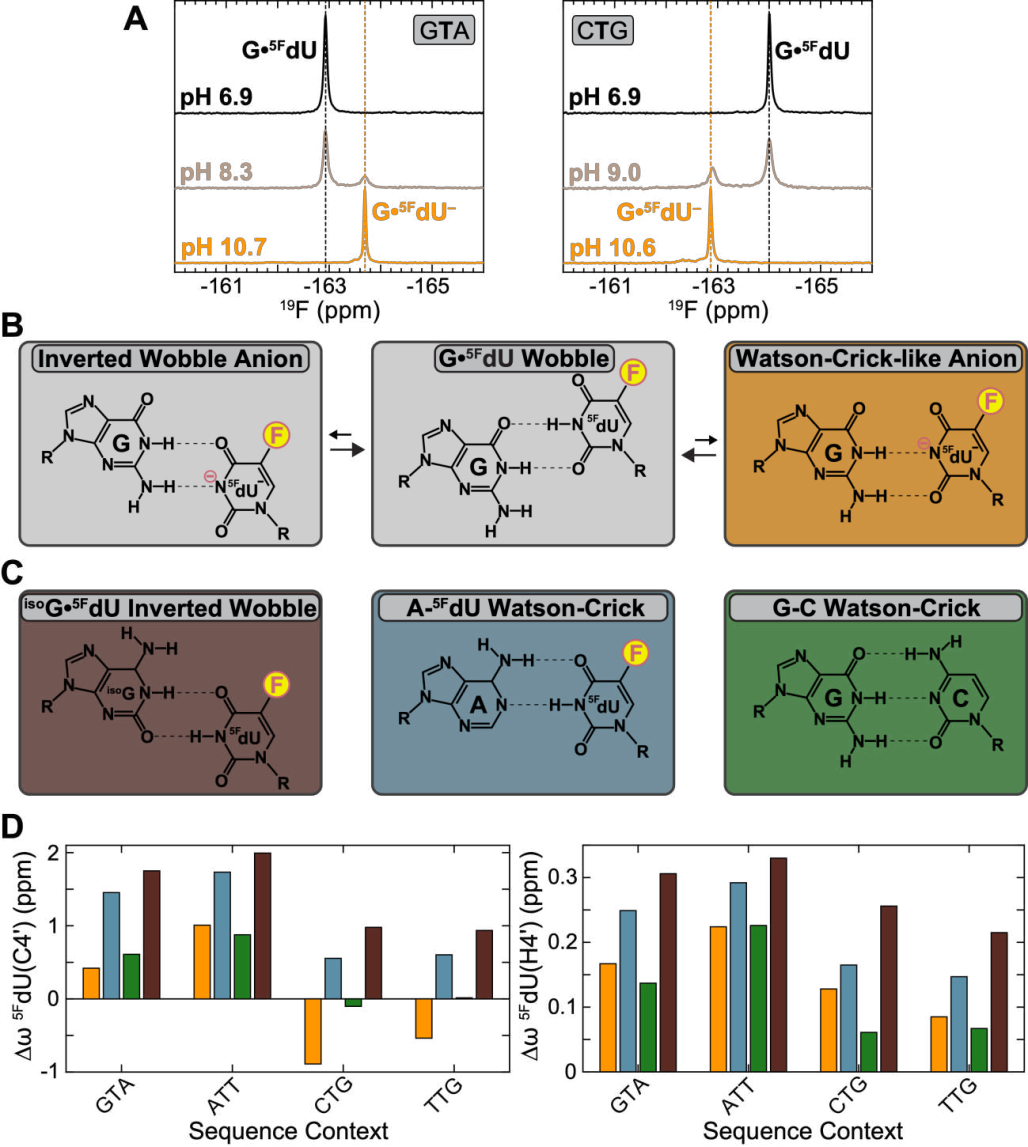
Detecting anionic WC-like G•^5F^dU^−^ via pH-dependent 1D ^19^F NMR. (A) Representative pH-dependent changes in 1D ^19^F NMR spectra showing the appearance of a resonance corresponding to WC-like G•^5F^dU^−^ and concomitant reduction of the intensity assigned to the G•^5F^dU wobble with increasing pH. (B) The G•^5F^dU mismatch may adopt an anionic Watson-Crick-like conformation (right), or an alternative anionic inverted wobble conformation formed by further displacement of uridine toward the minor groove beyond Watson-Crick alignment (left). (C) Using a chemical shift fingerprinting approach^82^ to rule out anionic inverted wobble conformation. ^iso^G•^5F^dU (^iso^G = isoguanosine) was used to mimic the inverted wobble and A-^5F^dU / G-C to mimic the Watson-Crick base pair^82, 86^. (D) Comparison of the U/T/C(C4’) and U/T/C(H4’) chemical shifts of G•^5F^dU^−^ against these mimics measured for four representative sequence contexts (GTA, ATT, CTG and TTG) at pH = 6.8 and T = 1 °C. Chemical shift differences Δω are relative to the wobble G•^5F^dU ground state. Additional NMR spectra and chemical shift perturbations (CSPs) are provided in Fig. S5.

To quantify the sequence-specific propensities to form the anionic WC-like G•^5F^dU^−^, we recorded pH-dependent 1D ^19^F NMR spectra at 1° C for all sixteen DNA duplexes containing the G•^5F^dU wobble. At pH ~6.9, a ^5F^dU(F5) resonance corresponding to the G•^5F^dU wobble ground state was observed (Fig. 3A, top), and its chemical shift varied ~2 ppm across sequence contexts, more than three times the ~0.6 ppm span of ^5F^dU(H3), highlighting the exquisite sensitivity of the ^19^F NMR nucleus (Fig. S4A).

Like the imino H3, the F5 chemical shift was primarily influenced by the 5’ neighbor but showed a weaker and opposite dependence, downfield for purines (Fig. S4A) and a smaller contribution from the 3’ neighbor, likely reflecting distinct ring current contributions at the F5 position (Supporting Discussion 1 and Fig. S4A). The chemical shifts also exhibited additivity (RMSD 0.34 ppm vs ~2 ppm span), comparable to imino proton resonances in both modified and unmodified duplexes (Fig. S4A).

Increasing the pH to ~8.0–8.3 resulted in the appearance of a second ^19^F resonance, consistent with a pH-dependent WC-like G•^5F^dU^−^ anionic species in slow exchange on the NMR chemical shift timescale (Fig. 3A, middle). The intensity of this resonance grew with pH while that of the wobble decreased, becoming dominant at pH >10.0 (Fig. 3A, bottom). Deprotonation was accompanied by sugar ^5F^dU(C1’) and ^5F^dU(C4’) CSPs, consistent with base de-shearing^81–82^ (Fig. S5).

The F5 chemical shift exhibited a unique sequence-dependence in the anionic state, shifting upfield with 5’-purine and downfield with pyrimidine, consistent a conformation distinct from that of the wobble (Fig. S4A). This chemical signature could not be explained solely by ring currents (see Supporting Discussion 1 and Fig. S4A) likely due to contributions from the anionic thymine base, a point we return to later (see Fig. S4B).

### Ruling out an anionic inverted wobble conformation

While we assigned the anionic state as WC-like G•^5F^dU^−^, we cannot rule out an alternative anionic inverted wobble formed by further displacement of uridine toward the minor groove beyond Watson-Crick alignment (Fig. 3B). This geometry has been observed in non-canonical contexts in DNA^27, 83^ and RNA^51–56^. In B-DNA, however, inverted wobble shearing is expected to be disfavored relative to the WC-like alignment^84^ and was previously ruled out as the anionic conformation detected by $R_{1\rho }$ NMR, since chemical shifts of the latter more closely matched that of the WC geometry^82, 85^. Nevertheless, the inverted wobble may predominate in other sequence contexts.

To test for an inverted wobble, we applied a chemical shift fingerprinting approach^82, 86^ to four representative sequences (GTA, ATT, CTG and TTG). We used ^iso^G•^5F^dU (^iso^G = isoguanosine) to mimic the inverted wobble and A-^5F^dU and G-C to mimic the Watson-Crick bp^82, 86^ (Fig. 3C, Fig. S5A). We then compared the U/T/C(C4’) chemical shifts of G•^5F^dU^−^, which can discriminate wobble versus inverted wobble geometries^82^ against these mimics. We generally avoid comparing base chemical shifts given contributions from base modification and ionization^82^.

In all cases, the ^5F^dU(C4’) and ^5F^dU(H4’) chemical shifts showed better agreement with the Watson-Crick A-^5F^dU or G-C relative to ^iso^G•^5F^dU (Fig. 3D). Although some deviations (<1 ppm) from the neutral Watson-Crick reference remained, these may reflect sequence-specific conformational adjustments to accommodate the anionic thymine base (Fig. S4B and Supporting Discussion 1). Other CSPs, including sugar protons, also supported a WC-like geometry (Fig. S5). While these results indicate that G•^5F^dU^−^ predominantly forms a WC-like conformation, the data cannot rule out a rapid equilibrium with a minor inverted wobble conformation^22, 84, 87^

### ^19^F NMR reveals dramatic sequence-specific propensities to form anionic G•^5F^dU^−^

**Figure 4.**
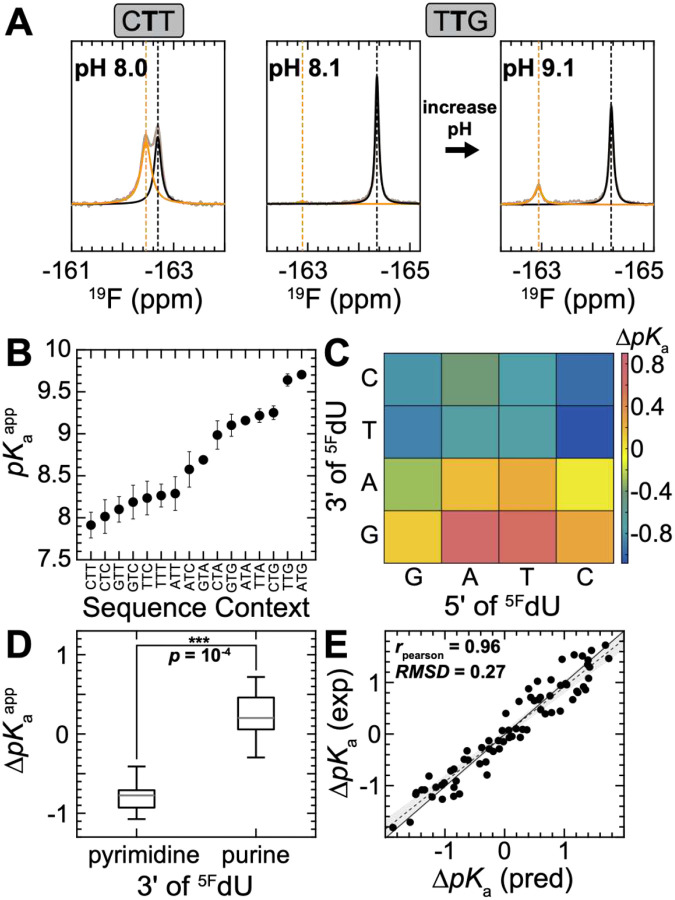
1D ^19^F NMR reveals dramatic sequence-dependent propensities for anionic WC-like G•^5F^dU^−^. (A) Representative examples of 1D ^19^F NMR spectra showing markedly different sequence-dependent propensities to form anionic WC-like G•^5F^dU^−^. (B) Apparent $pK_{a}(p{K_{a}}^{\text{app}})$ derived from ^19^F NMR measurements versus trinucleotide sequence context. The uncertainty was estimated by propagating the uncertainty from the Lorentzian line shape fits, which was obtained based on the square root of the diagonal values of the non-linear fitting covariance matrix. (C) Heatmap depicting the sequence-specific differences in $p{K_{\text{a}}}^{\text{app}}$ ($\Delta p{K_{a}}^{\text{app}}=p{K_{a}}^{\text{app}}(\text{seq})-p{K_{a}}^{\text{app}}(\text{ref})$; reference = CTA) with color-coded $\Delta pK_{a}$ values. (D) Whisker box plot of $\Delta pK_{a}$ for the two groups of 3’ neighbors of ^5F^dU. The division into two groups is statically significant based on Wilcoxon rank-sum test ($p=10^{-4}$). (E) Testing $p{K_{a}}^{\text{app}}$ additivity by comparing the change in $p{K_{a}}^{\text{app}}$ arising from changing both Watson-Crick neighbors with the sum of contributions from changing the individual 3’ and 5’ neighbors. The black line indicates the y=x line with slope one. Shown are fit to a linear model (black, dashed) with the region encompassing the 95% confidence intervals for slope and y-intercept shaded in grey, as well as the root-mean-square deviation (RMSD), and the Pearson correlation coefficient $\left(r_{pearson}\right)$.

The 1D ^19^F NMR experiment enabled the measurements of the WC-like G•^5F^dU^−^ population ($p_{\text{anion}}$) through integration of the anionic and wobble resonances robustly across all sixteen sequence contexts (Fig. 4A and Fig. S6, see methods). From $p_{\text{anion}}$ we determined the apparent $p{K_{a}}^{\text{app}}$ (Fig. 4B) using the Henderson-Hasselbalch equation:

$$pK_{a}^{\text{app}}=pH-\text{log}\left(\frac{p_{\text{anion}}}{1-p_{\text{anion}}}\right)$$

We observed a striking ~1.8-unit variation (~7.9 – 9.7) in $p{K_{a}}^{\text{app}}$, corresponding to 50-fold differences in the sequence-specific propensities to form the anionic state and energetic differences of $\Delta \Delta {G^{\circ }}_{\text{conf}}$ (anion) ~2.2 kcal/mol at physiological pH = 7.4. Thus, the anionic WC-like G•^5F^dU^−^ propensities are exquisitely sensitive to flanking Watson-Crick bps. Moreover, the $p{K_{a}}^{\text{app}}$ values clearly partitioned ($p=10^{-4}$) into two groups (Fig. 4C,D) depending on the 3’ neighbor of ^5F^dU: the anionic WC-like state was favored (lower $p{K_{a}}^{\text{app}}$) with a pyrimidine 3’ neighbor and disfavored (higher $p{K_{a}}^{\text{app}}$) with a purine 3’ neighbor. A similar trend appears in the limited $R_{1\rho }$ dataset for unmodified DNA^17^. Thus, the ^19^F NMR approach uncovered not only a pronounced but also distinctive conformational fingerprint for G•^5F^dU^−^.

Finally, our comprehensive measurement of sequence-specific $p{K_{a}}^{\text{app}}$s allowed us to test for energetic additivity, dissecting whether $p{K_{a}}^{\text{app}}$ shifts arising from changing the identify of both WC neighbors can be decomposed into the sum of the individual perturbations. Indeed, the $p{K_{a}}^{\text{app}}$ values were additive to within 0.27 $pK_{a}$ units (Fig. 4E), demonstrating that 5’ and 3’ contributions largely act independently.

### ^19^F NMR predicts sequence-specific propensity for unmodified G•T^−^

**Figure 5.**
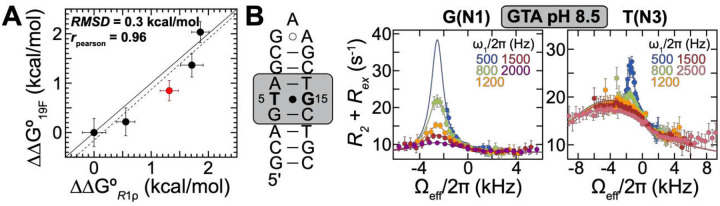
^19^F NMR predicts sequence-specific propensity for unmodified G•T^−^. (A) Correlation plot comparing the sequence-specific difference in free energy to form anionic WC-like G•T^−^ (ΔΔG=ΔG(seq)–ΔG(ref); using CTC as reference) measured using ^19^F NMR on G•^5F^dU versus ^15^N $R_{1\rho }$ relaxation dispersion on unmodified G•T sequences measured previously^17^ (black). An additional value measured in this study as a blind test for sequence context GTA is shown in red. The black line indicates the y=x line with slope one. Shown are the fit of the previously measured data to a linear model (black, dashed), as well as the root-mean-square deviation (RMSD), and the Pearson correlation coefficient $\left(r_{pearson}\right)$. The uncertainty in ${\Delta \Delta G}_{19F}$ was obtained by propagating the uncertainty in $p{K_{a}}^{\text{app}}$ (see Methods). The uncertainty in $\Delta \Delta G_{R1\rho }$ was obtained by propagating the uncertainty in the $R_{1\rho }$ data using a Monte Carlo procedure (see Methods). (B) Off-resonance ^15^N $R_{1\rho }$ profiles measured for G(N1) (left) and T(N3) (right) for an unmodified ^15^N isotopically labeled DNA with GTA sequence context measured at pH 8.5 and T = 4 °C. Spin-lock powers are color-coded. Error bars represent the experimental uncertainty of the $R_{1\rho }$ data and were obtained by propagating the uncertainty in $R_{1\rho }$ as described in Methods. The solid lines denote fits of the data to a 3-state exchange model with a triangular topology, using the Bloch-McConnell equations, as described in Methods.

Our approach assumes that the ^19^F modification uniformly shifts the $p{K_{a}}^{\text{app}}$ values so that the sequence-specific preferences for G•^5F^dU^−^ can be transferred to unmodified G•T^− 35^. We directly tested this assumption for F5 in four representative sequence contexts by comparing the conformational fingerprints for anionic WC-like G•^5F^dU^−^ (ΔΔG=ΔG(seq)-ΔG(ref); reference = CTC) measured by ^19^F NMR $\left(\Delta \Delta {G^{\circ }}_{19F}\right)$ with those measured previously^17^ for unmodified WC-like G•T^−^ $\left(\Delta \Delta {G^{\circ }}_{R1\rho }\right)$ using $R_{1\rho }$ on ^13^C/^15^N isotopically labeled DNA. The two measurements exhibited excellent agreement with RMSD = 0.3 kcal/mol, slope = 1.0 ± 0.2, and intercept = −0.2 ± 0.3 kcal/mol (Fig. 5A).

To further challenge the ^19^F NMR approach, we designed a blind test to address a gap in the available $R_{1\rho }$ data. We selected the GTA sequence context, which ^19^F NMR predicted to exhibit a distinct $\Delta \Delta {G^{\circ }}_{R1\rho }$ positioned near the midpoint of the overall sequence-dependent range, and for which no prior $R_{1\rho }$ measurements were available. To perform this test, we obtained an ^15^N isotopically labeled DNA sample containing the GTA context and performed ^15^N $R_{1\rho }$ relaxation dispersion measurements at high pH = 8.5 (Fig. 5B, Tables S2,S3). Indeed, the experimentally determined $\Delta \Delta {G^{\circ }}_{R1\rho }$ was centered relative to prior measurements and differed from the ^19^F-based prediction by ~0.5 kcal/mol (Fig. 5A, red), providing additional support for the accuracy and predictive power of the ^19^F NMR approach.

### Destabilizing interactions with anionic thymine determine sequence-dependence of G•^5F^dU^−^

**Figure 6.**
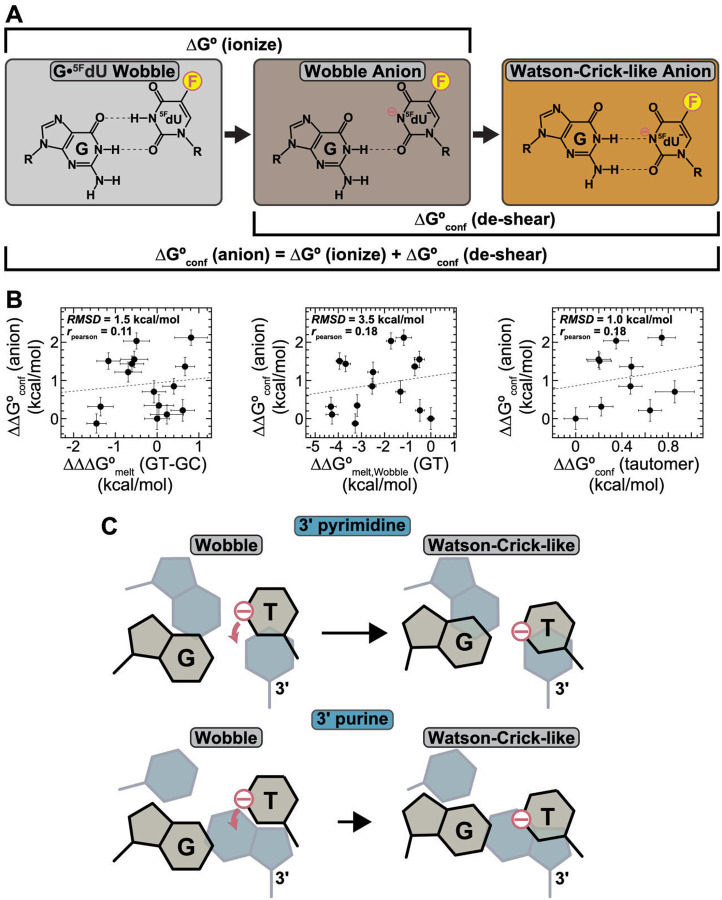
Destabilizing interactions with anionic thymine determine sequence-dependence of G•^5F^dU^−^. (A) The sequence-dependent $\Delta {G^{\circ }}_{\text{conf}}$ (anion) describing the energetics of the transition from wobble G•^5F^dU to anionic WC-like G•^5F^dU^−^ is decomposed into two energetic contributions assumed to be independent: pH-dependent deprotonation $\left(\Delta G^{\circ }(\text{ionize})\right)$ and base de-shearing $\left(\Delta {G^{\circ }}_{\text{conf}}(\text{de-shear},\text{anion})\right)$ to form the WC-like alignment. (B) Correlation plots for $\Delta \Delta {G^{\circ }}_{\text{conf}}(\text{anion})$, where ΔΔG=ΔG(seq)-ΔG(ref) using CTC as reference. $\Delta \Delta {G^{\circ }}_{\text{conf}}(\text{anion})$ is interpolated to pH 6.9 and compared against: $\Delta \Delta \Delta {G^{\circ }}_{\text{melt}}(GT-GC)=\Delta \Delta {G^{\circ }}_{\text{conf}}(\text{de-shear},\text{neutral})=\Delta \Delta {G^{\circ }}_{\text{melt},\text{Wobble}(\text{GT})}-\Delta \Delta {G^{\circ }}_{\text{melt},\text{Wobble}(\text{GC})}$ (left), $\Delta \Delta {G^{\circ }}_{\text{melt},\text{Wobble}(\text{GT})}$ (middle) and $\Delta \Delta {G^{\circ }}_{\text{conf}}$ (tautomer) (right). Shown are the fits to a linear model (black, dashed), as well as the root-mean-square deviations (RMSD), and the Pearson correlation coefficients $\left(r_{\text{pearson}}\right)$. The uncertainty in $\Delta \Delta {G^{\circ }}_{\text{conf}}(\text{anion})$ was obtained by propagating the uncertainty in $p{K_{a}}^{\text{app}}$ (see Methods). The uncertainties in $\Delta \Delta {G^{\circ }}_{\text{conf}}(\text{de-shear},\text{neutral})$ and $\Delta \Delta {G^{\circ }}_{\text{melt},\text{Wobble}(\text{GT})}$ were obtained by propagating the uncertainties from UV melting measurements. $\Delta \Delta {G^{\circ }}_{\text{conf}}(tautomer)$ was previously measured^17^ by ^15^N $R_{1\rho }$ relaxation dispersion at pH 6.9 and 25 °C. The uncertainty in $\Delta \Delta {G^{\circ }}_{\text{conf}}(tautomer)$ and was obtained by propagating the uncertainty in the previously measured $R_{1\rho }$ data using a Monte Carlo procedure (see Methods). (C) Forming the WC-like G•^5F^dU^−^ conformation requires displacement of the anionic ^5F^dU^−^ base toward its 3’ neighbor, making these sequence-specific destabilizing interactions strongly dependent on its identity, more destabilizing for purines than pyrimidines.

To dissect the sequence-dependence of $\Delta {G^{\circ }}_{\text{conf}}(\text{anion})$, we decomposed the transition from wobble G•^5F^dU to anionic WC-like G•^5F^dU^−^ into two energetic contributions assumed to be independent: pH-dependent deprotonation $\left(\Delta G^{\circ }(\text{ionize}\right)$) and base de-shearing ($\Delta {G^{\circ }}_{\text{conf}}(\text{de-shear},\text{anion})$) to form the WC-like alignment (Fig. 6A):

$$\Delta {G^{\circ }}_{\text{conf}}(\text{anion})=\Delta G^{\circ }(\text{ionize})+\Delta {G^{\circ }}_{\text{conf}}(\text{de-shear},\text{anion})$$

We first tested a simple thermodynamic model which assumes that both $\Delta G^{\circ }(\text{ionize})$ and any contribution to $\Delta {G^{\circ }}_{\text{conf}}(\text{de-shear},\text{anion})$ involving charge interactions with the anionic thymine base, are sequence independent. Under these assumptions:

$$\Delta \Delta G^{\circ }(\text{ionize})(\text{seq}-\text{ref})\approx 0$$

$$\Delta \Delta {G^{\circ }}_{\text{conf}}(\text{anion})(\text{seq}-\text{ref})\approx \Delta \Delta {G^{\circ }}_{\text{conf}}(\text{de-shear},\;\text{anion})(\text{seq}-\text{ref})\;\approx \Delta \Delta {G^{\circ }}_{\text{conf}}(\text{de-shear},\;\text{neutral})\text{seq}-\text{ref})$$

We estimated $\Delta \Delta {G^{\circ }}_{\text{conf}}(\text{de-shear},\;\text{neutral})$ using optical melting experiments across all 16 contexts, using G-C as a Watson-Crick proxy:

$$\Delta \Delta {G^{\circ }}_{\text{conf}}(\text{de-shear},\text{neutral})\approx \Delta \Delta {G^{\circ }}_{\text{melt},\text{Wobble}(\text{GT})}-\Delta \Delta {G^{\circ }}_{\text{melt},\text{WC}(\text{GC})}$$

Our estimate of $\Delta \Delta {G^{\circ }}_{\text{conf}}(\text{de-shear},\text{neutral})$ assumes that any sequence-dependent contributions from the loss of one hydrogen bond in G-C versus WC-like G•T^−^, or from substituting cytosine with thymine in the WC conformation, are negligible^35^.

Neither $\Delta \Delta {G^{\circ }}_{\text{conf}}(\text{de-shear},\text{neutral})$ (Fig. 6B), $\Delta \Delta {G^{\circ }}_{\text{melt},\text{Wobble}(\text{GT})}$ (Fig. 6B), or $\Delta \Delta {G^{\circ }}_{\text{melt},\text{WC}(\text{GC})}$ (data not shown) correlated with $\Delta \Delta {G^{\circ }}_{\text{conf}}(\text{anion})$. We also observed poor correlation with sequence-specific propensities to form the WC-like tautomers, G^enol^•T and G•T^enol^ (Fig. 6B)^17, 85, 88^. These deviations suggest that interactions involving the negative charge on the thymine base contribute to the unique sequence-dependence of $\Delta {G^{\circ }}_{\text{conf}}(\text{anion})$.

The charge can disrupt π–stacking, enhance electrostatic repulsion in the already negatively charged duplex, or create unfavorable anion–π interactions with electron-rich neighboring bases^89–93^. Such destabilizing effects can explain why, relative to monomers, the $p{K_{a}}^{\text{app}}$s of G•T and G•^5F^dU were consistently elevated by ~1–2 units, despite the expected energetic gains from restoring Watson-Crick alignment which should instead depress $p{K_{a}}^{\text{app}}$^94–95^ (note that h-bonds do not introduce a bias since there are two h-bonds in both WC-like G•T^−^ and wobble G•T). A similar ~1 unit increase relative to monomers has been observed for ^5F^dUTP and ^5Br^dUTP misincorporation opposite template G^28^ and for G(N1) in single-stranded DNA and RNA^96^, with stronger effects when G is flanked by purines versus pyrimidines. Computational work^89^ also shows that the stability of anionic thymine in DNA is strongly sequence-dependent, with neighboring purines less favorable than pyrimidines.

Forming the WC-like G•^5F^dU^−^ displaces the anionic ^5F^dU^−^ base toward its 3’ neighbor, making destabilization particularly sensitive to the 3’ neighbor identity, more destabilizing with purines, thus explaining the unique G•^5F^dU^−^ fingerprint (Fig. 6C). These destabilizing interactions may also induce conformational adjustments, such as displacing the 5’ purine toward the major groove, explaining the unique sequence-specific dependence of the ^5F^dU(F5) chemical shift in the anionic state (Fig. S4B and Supporting Discussion 1).

### Fingerprinting cancer mutational signatures using sequence-specific G•T^−^

We compared the unique WC-like G•T^−^ fingerprint against base substitution probabilities in the COSMIC database^36^ to identify potential mutational processes driven by this rare state. The similarity was quantified using the Jensen-Shannon divergence (JSD)^97^, which varies between 0 and 1 for maximum and minimum similarity, respectively.

**Figure 7.**
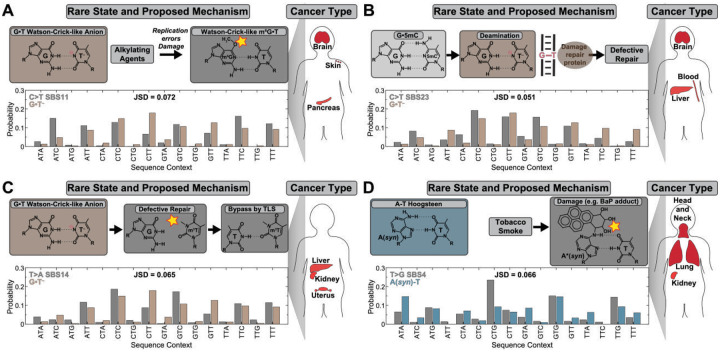
Conformational fingerprinting cancer mutational signatures. Shown are representative matches between the conformational fingerprints of (A-C) WC-like anionic G•T^−^ (in brown) and (D) A(syn)-T Hoogsteen base pair (in blue) with cancer mutational signatures (in grey) from the Catalogue of Somatic Mutations in Cancer (COSMIC) database^36^. Shown are along with their histogram correlation with the conformational fingerprints of rare mutagenic states. Shown for each match is a proposed mechanism for mutation driven by the rare mutagenic DNA state; comparison of the fingerprints and cancer signatures with the matching substitutions, and the cancer types linked to each signature. The similarities with conformational propensities were calculated using Jensen-Shannon divergence (JSD). C>T substitution from (A) SBS11 and (B) SBS23; (C) T>A substitution from SBS14; and (D) T>G substitution from SBS4.

WC-like G•T^−^ can promote formation and repair evasion of G•T mismatches leading to C>T or T>C substitutions^43, 98^. Accordingly, we compared the sequence-specific probabilities to form G•T^−^ with C>T or T>C substitution probabilities across 86 COSMIC mutational signatures of the Genome Reference Consortium Human genome build 37 (GRCh37)^36^. We observed strong (JSD ≤0.090) and statistically significant (p-value and false discovery rate <5%) similarity for C>T substitutions in several mutational signatures (Fig. 7A–B, Table S4), but none for T>C (see Supporting Discussion 2).

The SBS11 signature (JSD = 0.071, Fig. 7A) is dominated by C>T substitutions. It resembles the mutational spectrum of alkylating agents^3, 32^, and is associated with treatment with temozolomide (TMZ)^99–100^, which forms the O^6^-methylguanine (m^6^G) lesion^101^ (Fig. 7A). Interestingly, like WC-like G•T^−^, m^6^G drives mutagenesis by mispairing with thymine in the same WC-like geometry (Fig. 7A)^30, 102–103^. Normally, m^6^G is repaired by O^6^-methylguanine-DNA methyltransferase (MGMT), but in MGMT-deficient tumors it persists leading to C>T substitutions^32, 101, 104^. That our conformational fingerprinting analysis recovered a significant match to SBS11 is notable as most of >80 COSMIC mutational signatures bear no relation to WC-like geometries, making this association unlikely to be coincidental.

During replication, the wobble m^6^G•dCTP competes with WC-like m^6^G•dTTP mismatch, mirroring the wobble G•T⇌WC-like G•T^−^ equilibrium. Thus, the sequence-specificities could originate from m^6^G•dTTP misincorporation (Supporting Discussion 2). While the m^6^G driven replicative errors appear to be sequence-independent in *E. Coli*^37^, this may differ in human cancers. Alternatively, there is evidence that m^6^G formation by TMZ^101^ and other alkylation agents^105^ is dependent on sequence^37^, occurring more frequently when guanine is 5’-flanked by a purine i.e. opposing T base has a 3’ pyrimidine. The WC-like G•T^−^ might facilitate such alkylation damage by transiently exposing the guanine O^6^, normally h-bonded in the G•T wobble, thereby increasing its susceptibility to alkylation damage (Fig. 7A). Finally, we also cannot rule out that this match is coincidental.

Another match was observed for SBS23 (JSD = 0.051, Fig. 7B), dominated by C>T substitutions with unknown aetiology^106–107^. This signature appears mainly in exome sequencing datasets^106–107^, which are enriched in 5-methyl-cytosines (5mC)^108–110^. SBS23 could reflect 5mC deamination, which produces C>T but not T>C substitutions. WC-like G•T^−^ could form as a WC-like deamination intermediate before relaxing into the wobble state^111^ and/or promote repair evasion, both of which are sequence-dependent^98, 112–114^ (Fig. 7B). A related mechanism could explain the similarity with SBS3 (JSD = 0.080), associated with BRCA1/2-related homologous recombination deficiency^6, 115–117^ and CpG methylation in the BRCA1 promoter (Fig. S7A)^118^.

We did not observe a strong match with C>T and T>C substitutions for SBS14 (Table S4), a signature expected to isolate the contribution of nucleotide misincorporation since it arises under concurrent polymerase ε mutation and mismatch repair deficiency^3, 119^. This suggests that these C>T and T>C substitutions are not primarily driven by WC-like G•T^−^ misincorporation (Supporting Discussion 2). However, because SBS14 reflects conditions that favor persistence of G•T mismatches, we considered whether G•T^−^ could drive mutagenesis through m^3^T damage. In this pathway, G•T^−^ forms m^3^T, which acts as a TLS intermediate yielding mainly T>A with minor T>C and T>G substitutions^58^. Strikingly, comparison with all mutational signatures revealed a strong and statistically significant match (JSD = 0.065) with T>A substitutions specifically in SBS14^3, 119^ (Fig. 7C, Table S4). Although SBS14 is dominated by C>A, it also exhibits lower probability T>A substitutions, consistent with rare dual damage.

### Using conformational fingerprinting to assess contributions of other rare DNA states

Prior work measured sequence-specific propensities for other rare potentially mutagenic DNA conformations, including A(syn)-T and $G(\text{syn}){\text{-C}}^{+}$ Hoogsteen bps^23^ and A-T base opened state^35^.

We used our pipeline to compare these conformational fingerprints against the COSMIC database. No strong similarities were observed with the $G(\text{syn}){\text{-C}}^{+}$ Hoogsteen fingerprints, and while some matches were found with the A-T base-opening fingerprint, none were statistically significant.

In contrast, the A(syn)-T Hoogsteen fingerprint revealed strong (JSD ≤0.090) and statistically significant (p-value and false discovery rate <5%) similarities with several COSMIC signatures (Table S5). These numerous matches and their links to DNA damage, are consistent with both the relatively high abundance of A(syn)-T Hoogsteen conformation (~1%) and their demonstrated vulnerably to alkylation damage^32, 34^ generating lesions which if unrepaired can give rise to T>A, T>C, or T>G substitutions^58, 120–124^.

Compelling matches were SBS4 and SBS92, the two single-base substitution signatures associated with tobacco smoking^5, 125–128^. Both are attributed to benzo[a]pyrene (BaP), which primarily forms bulky guanine adducts causing C>A transversions but also adenine adducts yielding T>A, T>C and T>G substitutions^125, 128–130^. SBS4 and SBS92 closely matched A(syn)-T Hoogsteen propensities for T>A (JSD = 0.062, 0.025), T>C (0.061, 0.076), and T>G (JSD = 0.066 0.042) (Fig. 7D, Table S5). Similarly, the experimental BaP mutational spectrum in human tissue^128^ showed strong agreement with the NMR-derived A(syn)-T fingerprint (JSD < 0.075) (Table S6). Remarkably, the dominant BPDE-N^6^-dA adduct and related tobacco lesions^126^ such as εdA^131^ and m^1^A^58, 123, 132^ favor the *syn* conformation characteristic of the Hoogsteen pairing^133–138^, potentially facilitating adduct formation within B-DNA or promoting Hoogsteen-like purine-purine (A*-A, A*-G) intermediates during replication^136, 139^ that lead to T>A, T>C, and T>G transversions. A similar mechanism is known to be at play with the major guanine adduct which mispairs with adenine via a G*(syn)-A Hoogsteen conformation^140^.

A related mechanism may also account for the similarity with SBS22a (T>A, JSD = 0.081) and SBS22b^141–143^ (T>G, JSD = 0.082), both dominated by T>A substitutions linked to aristolochic acid exposure^125^. These result from bulky aristolactam-dA adducts, for which there is evidence that they favor the Hoogsteen conformation (Fig. S7B)^144–145^. In addition, the Hoogsteen fingerprint closely matched the experimental aristolochic acid mutational spectra measured in human cells^100, 146^ (Table S6). Additional matches (Table S5), including SBS42 associated with haloalkane exposure^147–148^, and SBS25 attributed to procarbazine chemotherapy^107, 149^ involve N-alkyl lesions^20, 150–153^. Although structural data for the resulting adenine adducts are lacking, modifications at N1^154^ and N6^155^ are plausible and could similarly favor the A(syn)conformation.

## Discussion

The notion that DNA itself contributes to replication errors challenges intuition: we typically blame mistakes on a faulty copying machine, not the sheet of paper. Yet, unlike letters on a page, DNA bps are dynamic, transiently sampling alternative conformations or sustaining damage. If captured by DNA polymerase at the wrong moment, these fleeting states can be misread, introducing mutations. Critically, because the probability of forming each mutagenic state depends on sequence context, they can leave their characteristic fingerprints in the genome, mutational signatures that record DNA’s own dynamic behavior. In this work, we test this concept directly, using conformational fingerprinting to connect rare DNA states measured *in vitro* to mutational processes observed *in vivo*.

The transition from wobble to WC conformation entails sub-angstrom displacement of the bases, yet we uncovered a striking 50-fold variation in propensity with sequence context. This pronounced sequence-dependence unveils a force originating from suboptimal interactions between the anionic thymine and its 3’ neighbor, demonstrating that loss of a single proton can profoundly shape the DNA energetic landscape. The implications extend beyond the sequence-specificity of replication errors to include other processes that can act on this anionic state, from transcriptional^156^ and translational^157^ errors to CRISPR-Cas off-target editing^158^. In this regard, our findings clarify prior observations^156^ in RNA:DNA hybrids, where WC-like G•U^−^ forms 10-fold more readily than G•T^−^: while a 4-fold difference can be attributed to uridine’s lower $pK_{a}$, our ^19^F NMR results reveal an additional 3-fold contribution from sequence context (TTC versus GTA). Comparably strong sequence effects might arise with other mutagenic states involving cationic bases^20, 35, 159^.

We also find that the G•T^−^ anion may play a more significant role in G•T misincorporation than previously appreciated. While misincorporation has traditionally been attributed to the more abundant WC-like tautomers^17^ G^enol^•T and G•T^enol^, we find that in certain sequence contexts such as CTT, CTC, GTT, GTC, and TTC and at pH >7.4, G•T^−^ achieves populations comparable to or even exceeding the measured populations of tautomeric states (Fig. S8). Determining the contributions of WC-like states to replicative errors linked to C>T and T>C substitutions will require measuring sequence-specific propensities, including conformational kinetics for both tautomeric and anionic states of G•T and also for other contributing mismatches such as A•C.

Finally, by comparing conformational fingerprints to the COSMIC catalogue, we identified several mutational signatures associated with exposure to damaging agents and therapies that may be driven by WC-like G•T^−^ anion and A(syn)-T Hoogsteen bps. These connections provide concrete hypotheses, such as links to alkylation and deamination that need to be verified experimentally. Thus, our results highlight conformational fingerprinting as a framework for bridging molecular biophysics and genomic epidemiology, offering a path to explore how the dynamic properties of DNA might shape genome stability and influence human disease.

## Materials and Methods

### Sample Preparation

#### Unlabeled oligonucleotides.

All unmodified DNA (~2 mg) oligonucleotides were purchased from Integrated DNA Technologies (IDT) with standard desalting purification. These include sixteen unlabeled oligonucleotides 5’-GCAZ**T**WGCGAAGCW’**G**Z’TGC-3’ (Z-Z’ and W-W’ = A-T, T-A, G-C and C-G) corresponding to the hairpins with G•T mismatches, four oligonucleotides corresponding to hairpins with trinucleotide sequence contexts GTA, ATT, CTG and TTG with a central G-C Watson-Crick bp, and 16 × 2 DNA oligonucleotides 5’-CAGCAZ**(C/T)**WGCGC-3’ and 5’-GCGCW’**G**Z’TGCTG-3’ (Z-Z’ and W-W’ = A-T, T-A, G-C and C-G) corresponding to the DNA duplexes used in the UV melting experiments (Table S1).

#### ^5F^dU-modified oligonucleotides.

All chemically modified oligonucleotides (~2 mg) containing 5FdU and ^iso^G used for NMR studies were purchased from Yale Keck Oligonucleotide Synthesis with cartridge purification. These include sixteen 5’-GCAZ^**5F**^**dU**WGCGAAGCW’**G**Z’TGC-3’ (Z-Z’ and W-W’ = A-T, T-A, G-C and C-G) (Table S1), four single-stranded DNA oligonucleotides with trinucleotide sequence contexts GTA, ATT, CTG and TTG containing a central A-^5F^dU Watson-Crick reference, and another four with trinucleotide sequence contexts GTA, ATT, CTG and TTG containing a central ^iso^G•^5F^dU mismatch (Table S1).

#### ^15^N-labeled oligonucleotides.

A residue-specifically (T5 and G15) ^15^N-site-labeled single-stranded DNA oligonucleotide (~2 mg), corresponding to the hairpin with a GTA sequence context, with the sequence 5’-GCAG**T**AGCGAAGCT**G**CTGC-3’, was purchased from Yale Keck Oligonucleotide Synthesis Facility with cartridge purification.

#### NMR Buffers.

The NMR buffer consisted of 15 mM sodium phosphate, 25 mM sodium chloride and 0.1 mM EDTA in 90% H_2_O:10%D_2_O at varying pH. The pH was adjusted using sodium hydroxide (NaOH), while keeping the sodium ion concentration constant at 25 mM.

#### Preparation of NMR samples.

All the DNA hairpin samples (~10–20 μM) were annealed in water by heating to 95 °C for 5–8 min followed by fast cooling on ice for ~20 minutes. Samples were then exchanged into the NMR buffer using Amicon Ultra-15 centrifugal concentrators (3-kDa cutoff, Millipore Sigma) to a final nucleic acid concentration of 300 μM – 1 mM. Extinction coefficients used to measure oligonucleotide sample concentrations were determined using the ADT Bio Oligo Calculator (https://www.atdbio.com/tools/oligo-calculator). The sample pH was measured using a thin pH electrode.

### NMR Experiments

1D ^19^F NMR experiments were performed on a 500 MHz Bruker Avance III HD spectrometer equipped with a 5 mm cryogenic probe (QCI 500 H&F-P/C/N-D-5-Z). All other NMR experiments were performed on Bruker Avance III 500 MHz, 600 MHz or 700 MHz spectrometers equipped with HCN cryogenic probes. NMR experiments were carried out at 1 °C unless stated otherwise. Data were processed using NMRpipe^160^ and analyzed using SPARKY^161^.

#### Resonance assignments.

The DNA imino resonances for all 16 unmodified (G•T) and 9 modified (G•^5F^dU) hairpin constructs at pH 6.8–6.9 were assigned using 2D ^1^H-^1^H NOESY (mixing time = 175 ms), collected at 600 MHz (Fig. S2). The assignment of the imino resonances for the rest of the modified hairpin constructs was transferred from its unmodified counterpart. 2D ^1^H-^13^C HSQC experiments for select sequences, including three unmodified (GTA, ATT and CTG) and four F5 modified (GTA, ATT, CTG and TTG) hairpins were also measured at 600 MHz, and were used in combination with the corresponding 2D ^1^H-^1^H NOESY experiments to assign the aromatic (H6C6/H8C8/H2C2) and sugar (H1’C1’/H4’C4’) protons and carbons. Resonance assignment was also performed at high pH >10.0 for four select hairpins (GTA, ATT, CTG and TTG) containing a G•^5F^dU mismatch.

#### pH-dependent NMR experiments for measuring WC-like G•^5F^dU^−^ propensities.

pH-dependent 1D ^1^H and ^19^F NMR spectra were collected for all the 16 ^5F^dU-modified hairpins. Both the ^5F^dU(H3) and G(H1) imino resonances disappeared at pH >10.0 due to deprotonation of ^5F^dU(H3) and G(H1) and increased base catalyzed exchange under these basic conditions.

#### Assessing inverted wobble conformation.

1D ^1^H and 2D NOESY and HSQC spectra were collected for the four hairpins containing either the inverted wobble (^iso^G•^5F^dU) or the Watson-Crick (A-^5F^dU or G-C) mimics at pH 6.8 (Fig. 3C). The ^iso^G•^5F^dU mismatch adopted a non-Watson-Crick conformation, as indicated by the ^1^H imino chemical shifts of ^5F^dU(H3) and ^iso^G(H1) at ~ 12 ppm and ~ 10 ppm, respectively, with all other residues forming canonical Watson-Crick bps, as reported previously^82^ (Fig. S5A). The ^1^H imino spectra also showed pronounced broadening of the ^5F^dU(H3) and ^iso^G(H1) resonances, which were 0.3 – 0.7 ppm downfield shifted relative to the G•^5F^dU wobble as expected^82^. In contrast, the A-^5F^dU bp in the Watson-Crick reference duplex adopted a canonical Watson-Crick conformation, as evidenced by the ^5F^dU(H3) resonance at ~ 14 – 14.5 ppm and diagnostic NOE cross peaks such as ^5F^dU(H3)···A(H2) (Fig. S5A). As reported previously^78–79^, the ^5F^dU(H3) imino proton resonance was significantly broadened.

#### ^15^N $R_{1\rho }$ relaxation dispersion.

Off-resonance ^15^N $R_{1\rho }$ relaxation dispersion (RD) experiments^39–40^ were performed on a Bruker Avance III 700 MHz spectrometer equipped with a 5 mm cryogenic probe (TCI 700 H&F-C/N-D-05 Z) at 4 °C, and implemented using a 1D selective excitation scheme as described in prior studies^162–163^. The spin-lock powers $\left(\omega_{1}/2\pi \right)$ ranged from 500 to 2000 Hz for G(N1) or 2500 Hz for T(N3), while the offsets ranged from ±3.5 times the spin-lock power, respectively (Table S2). For each resonance, five delay times were used during the relaxation period with maximum duration of 120 ms for G(N1) and 72 ms for T(N3).

#### Analysis of ^15^N $R_{1\rho }$ data.

The ^15^N $R_{1\rho }$ data was analyzed as described previously^85^. Briefly, peak were extracted using NMRPipe^160^, and fitted to a mono-exponential decay as a function of the delay time to obtain the $R_{1\rho }$ value for each spin-lock power and offset combination. The uncertainty in $R_{1\rho }$ was estimated using a Monte Carlo procedure^40^. The $R_{1\rho }$ data was fit to a threestate exchange model with triangular topology using the Bloch-McConnell equations^164^, to extract exchange parameters, including the populations ($p_{ES1}$ and $p_{ES2}$) of the excited states ES1 = tautomer and ES2=anion; the exchange rates between ESs and the ground state (GS) ($k_{ex,GS:ES1};k_{ex,GS:ES2};k_{ex,ES1:ES2},in$ which $k_{ex}=k_{\text{forward}}+k_{\text{backward}}$); the difference between the chemical shifts of each ESs and the GS $\left(\Delta \omega_{ES1}=\omega_{ES1}-\omega_{GS};\Delta \omega_{ES2}=\omega_{ES2}-\omega_{GS}\right)$; and the transverse $\left(R_{2}\right)$ and longitudinal $\left(R_{1}\right)$ relaxation rates. In all cases, it was assumed that $R_{2,GS}=R_{2,ES1}=R_{2,ES2}=R_{2}$ and $R_{1,GS}=R_{1,ES1}=R_{1,ES2}=R_{1}$. Combined fits of the $R_{1\rho }$ data for multiple nuclei were performed by sharing $p_{ES1}$, $p_{ES2}$, $k_{ex,GS:ES1}$, $k_{ex,GS:ES2}$, and $k_{ex,ES1:ES2}$.

The initial alignment of magnetization during the Bloch-McConnell simulations was determined based on the $k_{ex}/\Delta \omega$ ratio of the majorly populated ES, as described previously^40^. The uncertainty in the exchange parameters was obtained using a Monte-Carlo scheme as described previously^165^. The fitted exchange parameters are summarized in Table S3.

Off-resonance $R_{1\rho }$ profiles were generated by plotting $\left(R_{2}+R_{ex}\right)=\left(R_{1\rho }-R_{1}{\text{cos}}^{2}\theta \right)/{\text{sin}}^{2}\theta$, where θ is the angle between the effective field of the observed resonance and the z-axis, as a function of $\Omega_{\text{eff}}=\omega_{\text{obs}}-\omega_{\text{RF}}$, in which $\omega_{\text{obs}}$ is the Larmor frequency of the observed resonance and $\omega_{\text{RF}}$ is the angular frequency of the applied spin-lock. The uncertainty in $\left(R_{2}+R_{ex}\right)$ was determined by propagating the uncertainty in $R_{1\rho }$ obtained as described above.

### Determining $p{K_{a}}^{\text{app}}$ from pH-dependent NMR

pH-dependent 1D ^19^F NMR spectra were processed using a shifted sine bell function and automatic base-line correction. Spectra were analyzed using an in-house script utilizing the non-linear least-squares curve Matlab^®^ fitting function to fit the spectral peaks to either one, two or three Lorentzian peaks. The relative populations of the wobble G•^5F^dU $\left(p_{\text{wobble}}\right)$ and anionic $\left(p_{\text{anion}}\right)$ G•^5F^dU^−^, and in some cases the third resonance, were calculated from the ratio of the integrals of the fitted Lorentzian peaks. $p_{\text{anion}}$ was then used to calculate the sequence-dependent apparent $p{K_{a}}^{\text{app}}$ using the Henderson-Hasselbalch equation:

$$pK_{a}^{\text{app}}=pH-\text{log}\left(\frac{p_{\text{anion}}}{1-p_{\text{anion}}}\right)$$

Similar $p{K_{a}}^{\text{app}}$ values were obtained from pH-dependent measurements for the GTA and TTT DNA duplexes, validating the approach (Fig. S6B).

All $p{K_{a}}^{\text{app}}$ values were obtained from measurements at 1 °C. Similar $p{K_{a}}^{\text{app}}$ values (ΔΔG<0.2kcal/mol) and sequence-specific energetics (ΔΔG<0.06kcal/mol for G•^5F^dU and <0.04 kcal/mol for G•T^17^) were obtained at varying temperatures (5 °C – 20 °C) for two sequence contexts CTC and ATC (Fig. S6C).

### Chemical shift and $p{K_{a}}^{\text{app}}$ additivity

The NMR chemical shifts and the $p{K_{a}}^{\text{app}}$ values exhibited strong variations when altering the 5’ and 3’ neighbors of the G•T and G•^5F^dU mismatch. We asked whether the perturbations relative to a reference sequence context (e.g. GTA) arising from changing the identify of both neighbors (e.g. GTA→CTG) can be decomposed into the sum of the individual perturbations arising from changing either the 5’ (GTA→CTA) or 3’ neighbor (GTA→GTG)^23^:

GTA→CTG=GTA→CTA+GTA→GTG

$$\Delta {\omega^{\text{pred}}}_{5’+3’}=\Delta {\omega^{\text{exp}}}_{5’}+\Delta {\omega^{\text{exp}}}_{3’}$$

$$\Delta p{{K_{\text{a}}}^{\text{pred}}}_{5’+3’}=\Delta p{{K_{\text{a}}}^{\text{exp}}}_{5’}+\Delta p{{K_{\text{a}}}^{\text{exp}}}_{3’}$$

Thus, we compared $\Delta {\omega^{\text{pred}}}_{5’+3’}=\Delta {\omega^{\text{exp}}}_{5’}+\Delta {\omega^{\text{exp}}}_{3’}$ and $\Delta p{{K_{\text{a}}}^{\text{pred}}}_{5’+3’}=\Delta p{{K_{\text{a}}}^{\text{exp}}}_{5’}+\Delta p{{K_{\text{a}}}^{\text{exp}}}_{3’}$ to the values measured experimentally $\Delta {\omega^{\text{exp}}}_{5’+3’}$ and $\Delta p{{K_{\text{a}}}^{\text{pred}}}_{5’+3’}$ across the 72 independent mutational cycles^23^ (Figs 4E, S3B,C, S4A).

### UV melting experiments

UV melting experiments were performed on DNA duplexes (3μM) in the NMR buffer. To optimize melting experiments, the duplexes were elongated by adding 5’-CA-3’ and 5’-GC-3’ at the terminal ends on the 5’ and 3’ side of C/T, respectively (5’-CAGCAZ**(C/T)**WGCGC-3’ and 5’-GCGCW’**G**Z’TGCTG-3’). One sequence context (TTC) was excluded due to lack of reproducible measurements across replicates.

Melting experiments were performed as described previously^35^. The absorbance at 260 nm (A_260_) was monitored during heating from 15 to 95 °C at a rate of 1.0 °C/min on a PerkinElmer Lambda 25 UV/VIS spectrometer (PerkinElmer, USA) with a 400μL sample in a Teflon-stoppered quartz cell (path length = 1 cm). The melting temperature (T_m_) and standard enthalpy change of melting $\left(\Delta H^{\circ }\right)$ were determined by fitting A_260_ as a function of temperature (T, in Kelvin):

$$A_{260}(T)=\left(m_{ss}T+b_{ss}\right)\times p_{ss}+\left(m_{ds}T+b_{ds}\right)\times p_{ds}$$

$$p_{ss}=1-\frac{1+4e^{-\left(\frac{1}{T_{m}}-\frac{1}{T}\right)\frac{\Delta H^{\circ }}{R}}-\sqrt{1+8e^{-\left(\frac{1}{T_{m}}-\frac{1}{T}\right)\frac{\Delta H^{\circ }}{R}}}}{4e^{-\left(\frac{1}{T_{m}}-\frac{1}{T}\right)\frac{\Delta H^{\circ }}{R}}}$$

$$p_{ds}=1-p_{ss}$$

where m_ss_, b_ss_, m_ds_, and b_ds_ are coefficients describing the temperature dependence of molar extinction coefficients for single strands (ss) and double strand (ds), respectively, p_ss_ and p_ds_ are the populations of single strands and double strand, respectively, and R is the gas constant 1.987 cal/mol/K. The standard entropy change $\left(\Delta S^{\circ }\right)$ was computed using:

$$\Delta S^{\circ }=\frac{\Delta H^{\circ }}{T_{m}}-Rln\frac{C_{\text{total}}}{2}$$

where C_total_ is the total concentration of duplex DNA (i.e., 3μM). The standard free energy change for duplex hybridization $\left(\Delta G^{\circ }\right)$ was computed using: $\Delta G^{\circ }=\Delta H^{\circ }-T\Delta S^{\circ }.{\Delta G^{\circ }}_{25}$ at 25°C was computed using:

$$\Delta G_{25}^{\circ }=\Delta H^{\circ }-(25+273.15)\Delta S^{\circ }$$

The mean value and uncertainty in T_m_, $\Delta H^{\circ }$, $\Delta S^{\circ }$, and ${\Delta G^{\circ }}_{25}$ were set to the average and standard deviation from multiple measurements (≥3).

### Pipeline for fingerprinting cancer mutational signatures

We used an in-house Matlabfi program to calculate the Jensen-Shannon divergence (JSD)^97, 166–167^ between base substitutions (C>T, T>C, C>A, C>G, T>A, T>G) from cancer mutational signatures available in the COSMIC database of the Genome Reference Consortium Human genome build 37 (GRCh37)^36^ and the NMR-derived sequence-specific conformational propensities. The Matlab^®^ program and a python version used for JSD fingerprinting are available on GitHub at https://github.com/alhashimilab/DNA-conformational-fingerprinting.git (permanent DOI: 10.5281/zenodo.17504626).

Conformational fingerprints were generated from the conformational propensities (of anionic WC-like G•T^−^, A(syn)-T or $G(\text{syn}){\text{-C}}^{+}$ Hoogsteen bps, and A-T opened states) by normalizing the available propensities across sequence contexts. We then compared each conformational fingerprint with base substitutions which may involve the formation of the rare DNA conformational state.

The A(syn)-T Hoogsteen bp exposes the Watson-Crick face of adenine to alkylation damage generating lesions such as m^1^A, which via TLS can yield T>A, T>C, or T>G substitutions (i.e. A-T → m^1^A-T → m^1^A•A → T-A; A-T → m^1^A-T → m^1^A•C → G-C; and A-T → m^1^A-T → m^1^A•G → C-G). We therefore compared the A(*syn*)-T Hoogsteen bp fingerprint to T>A, T>C, or T>G substitutions. Similarly, the $G(\text{syn}){\text{-C}}^{+}$ Hoogsteen exposes the guanine to alkylation damage generating lesions such as m^1^G, which via TLS can yield C>A, C>T and C>G substitutions (i.e. G-C → m^1^G-C → m^1^G•A → T-A; G-C → m^1^G-C → m^1^G•T → A-T; and G-C → m^1^G-C → m^1^G•G → C-G). We therefore compared the $G(\text{syn}){\text{-C}}^{+}$ Hoogsteen conformational fingerprint to C>A, C>T, or C>G signatures. The A-T opened state may be an intermediate in mutations involving T>A or T>G substitutions (i.e. A-T → A T_open_ → C•T_open_ → C-G; A-T → A T_open_ → T•T_open_ → T-A). We therefore compared the A-T opened state conformational fingerprint to T>A and T>G substitutions.

For each comparison, sequence-specific substitution probabilities were normalized and their JSD relative to the sequence-specific conformational propensities was calculated. The statistical significance of a given match was assessed by randomly sampling substitution probabilities from all possible substitutions across all signatures, generating a null distribution (n = 10^6^ times). This random scheme was chosen because it is sampling a probability of a certain mutation in a given sequence context within the distribution of all possible probabilities across all signatures. JSD values were computed for each random draw, and p-values and false discovery rates (FDR) were then derived from this distribution. In the case of A(syn)-T, $G(\text{syn}){\text{-C}}^{+}$, and base opened states, for which less than 16 propensities were available, the comparison was made for only those available sequence contexts. Mutational propensities for missing sequence contexts were omitted from the comparison, and the remaining propensities were normalized and used in the pipeline. JSD values ≤0.090 with both p-value and FDR <5% were considered strong similarities.

## Supplementary Material

Supplementary Data

Supplementary Figures S1–S8 and Supplementary Tables S1–S6 are available online

Supplementary Files

This is a list of supplementary files associated with this preprint. Click to download.

- SzekelyExcitedCancerStatesSI.pdf

## Data Availability

A public git repository is available at https://github.com/alhashimilab/DNA-conformational-fingerprinting.git (permanent DOI: 10.5281/zenodo.17504626) containing all source code and a minimum dataset sufficient to reproduce Figures 2–7, S1–S8 and all supplementary tables and statistics in this manuscript. Full data sets are available upon request.

## References

[1] Lupski J. R., , Clan genomics and the complex architecture of human disease. Cell 2011, 147 (1), 32–43. 21962505 10.1016/j.cell.2011.09.008 PMC3656718

[2] Acuna-Hidalgo R., , New insights into the generation and role of de novo mutations in health and disease. Genome Biol 2016, 17 (1), 241. 27894357 10.1186/s13059-016-1110-1 PMC5125044

[3] Alexandrov L. B., , Signatures of mutational processes in human cancer. Nature 2013, 500 (7463), 415–421. 23945592 10.1038/nature12477 PMC3776390

[4] Zhao E. Y., , Whole-Genome Sequencing in Cancer. Cold Spring Harbor Perspectives in Medicine 2019, 9 (3). 10.1101/cshperspect.a034579 PMC6396343 29844223

[5] Alexandrov L. B., , The repertoire of mutational signatures in human cancer. Nature 2020, 578 (7793), 94–101. 32025018 10.1038/s41586-020-1943-3 PMC7054213

[6] Nik-Zainal S., , Mutational Processes Molding the Genomes of 21 Breast Cancers. Cell 2012, 149 (5), 979–993. 22608084 10.1016/j.cell.2012.04.024 PMC3414841

[7] Alexandrov Ludmil B., , Deciphering Signatures of Mutational Processes Operative in Human Cancer. Cell Reports 2013, 3 (1), 246–259. 23318258 10.1016/j.celrep.2012.12.008 PMC3588146

[8] Lawrence M. S., , Mutational heterogeneity in cancer and the search for new cancer-associated genes. Nature 2013, 499 (7457), 214–218. 23770567 10.1038/nature12213 PMC3919509

[9] Pfeifer G. P., , Tobacco smoke carcinogens, DNA damage and p53 mutations in smoking-associated cancers. Oncogene 2002, 21 (48), 7435–7451. 12379884 10.1038/sj.onc.1205803

[10] Alexandrov L. B., , Mutational signatures associated with tobacco smoking in human cancer. Science 2016, 354 (6312), 618–622. 27811275 10.1126/science.aag0299 PMC6141049

[11] Sanchez A. M., , Initiation of repair of A/G mismatches is modulated by sequence context. DNA Repair 2003, 2 (8), 863–878. 12893083 10.1016/s1568-7864(03)00067-3

[12] Helleday T., , Mechanisms underlying mutational signatures in human cancers. Nature Reviews Genetics 2014, 15 (9), 585–598. 10.1038/nrg3729 PMC6044419 24981601

[13] Hu X., , Characteristics of mutational signatures of unknown etiology. NAR Cancer 2020, 2 (3). 10.1093/narcan/zcaa026 PMC7520824 33015626

[14] Oman M., , How Sequence Context-Dependent Mutability Drives Mutation Rate Variation in the Genome. Genome Biology and Evolution 2022, 14 (3). 10.1093/gbe/evac032 PMC8920511 35218359

[15] Steele C. D., , An overview of mutational and copy number signatures in human cancer. The Journal of Pathology 2022, 257 (4), 454–465. 35420163 10.1002/path.5912 PMC9324981

[16] Nair D. T., , Replication by human DNA polymerase-ι occurs by Hoogsteen base-pairing. Nature 2004, 430 (6997), 377–380. 15254543 10.1038/nature02692

[17] Kimsey I. J., , Dynamic basis for dG•dT misincorporation via tautomerization and ionization. Nature 2018, 554 (7691), 195–201. 29420478 10.1038/nature25487 PMC5808992

[18] Bouchal T., , Importance of base-pair opening for mismatch recognition. Nucleic Acids Res 2020, 48 (20), 11322–11334. 33080020 10.1093/nar/gkaa896 PMC7672436

[19] Buchel G., , Structural basis for DNA proofreading. Nature Communications 2023, 14 (1), 8501. 10.1038/s41467-023-44198-8 PMC10752894 38151585

[20] Gu S., , Dynamic basis for dA•dGTP and dA•d8OGTP misincorporation via Hoogsteen base pairs. Nat Chem Biol 2023, 19 (7), 900–910. 37095237 10.1038/s41589-023-01306-5 PMC13290353

[21] Mishra S. K., , The role of nucleotide opening dynamics in facilitated target search by DNA-repair proteins. Biochimica et Biophysica Acta (BBA) - Gene Regulatory Mechanisms 2024, 1867 (2), 195026. 38641240 10.1016/j.bbagrm.2024.195026

[22] V gele J., , Structure of an internal loop motif with three consecutive U•U mismatches from stem–loop 1 in the 3′-UTR of the SARS-CoV-2 genomic RNA. Nucleic Acids Res 2024, 52 (11), 6687–6706. 38783391 10.1093/nar/gkae349 PMC11194097

[23] Manghrani A., , Quantitative and Systematic NMR Measurements of Sequence-Dependent A–T Hoogsteen Dynamics in the DNA Double Helix. Biochemistry 2025, 64 (5), 1042–1054. 39982856 10.1021/acs.biochem.4c00820

[24] Bebenek K., , Replication infidelity via a mismatch with Watson-Crick geometry. PNAS 2011, 108 (5), 1862–1867. 21233421 10.1073/pnas.1012825108 PMC3033279

[25] Wang W., , Structural evidence for the rare tautomer hypothesis of spontaneous mutagenesis. PNAS 2011, 108 (43), 17644–17648. 22006298 10.1073/pnas.1114496108 PMC3203791

[26] Koag M.-C., , The spontaneous replication error and the mismatch discrimination mechanisms of human DNA polymerase β. Nucleic Acids Res 2014, 42 (17), 11233–11245. 25200079 10.1093/nar/gku789 PMC4176172

[27] Xia S.; Konigsberg W. H., Mispairs with Watson-Crick base-pair geometry observed in ternary complexes of an RB69 DNA polymerase variant. Protein Science 2014, 23 (4), 508–513. 24458997 10.1002/pro.2434 PMC3970900

[28] Yu H., , Ionization of bromouracil and fluorouracil stimulates base mispairing frequencies with guanine. J Biol Chem 1993, 268 (21), 15935–15943. 7688001

[29] Kunkel T. A.; Bebenek K., DNA Replication Fidelity. Annual Review of Biochemistry 2000, 69 (1), 497–529. 10.1146/annurev.biochem.69.1.497 10966467

[30] Warren J. J., , The structural basis for the mutagenicity of O^6^-methyl-guanine lesions. PNAS 2006, 103 (52), 19701–19706. 17179038 10.1073/pnas.0609580103 PMC1750904

[31] Basu A. K., , Translesion Synthesis of 2’-Deoxyguanosine Lesions by Eukaryotic DNA Polymerases. Chemical Research in Toxicology 2017, 30 (1), 61–72. 27760288 10.1021/acs.chemrestox.6b00285 PMC5241707

[32] Basu A. K.; Essigmann J. M., Establishing Linkages Among DNA Damage, Mutagenesis, and Genetic Diseases. Chemical Research in Toxicology 2022, 35 (10), 1655–1675. 35881568 10.1021/acs.chemrestox.2c00155 PMC10201539

[33] Buisson R., , Passenger hotspot mutations in cancer driven by APOBEC3A and mesoscale genomic features. Science 2019, 364 (6447), eaaw2872. 31249028 10.1126/science.aaw2872 PMC6731024

[34] Xu Y., , Hoogsteen base pairs increase the susceptibility of double-stranded DNA to cytotoxic damage. J Biol Chem 2020, 295 (47), 15933–15947. 32913127 10.1074/jbc.RA120.014530 PMC7681010

[35] Rangadurai A., , Measuring thermodynamic preferences to form non-native conformations in nucleic acids using ultraviolet melting. PNAS 2022, 119 (24), e2112496119. 10.1073/pnas.2112496119 PMC9214542 35671421

[36] Tate J. G., , COSMIC: the Catalogue Of Somatic Mutations In Cancer. Nucleic Acids Res 2018, 47 (D1), D941–D947. 10.1093/nar/gky1015 PMC6323903 30371878

[37] Delaney J. C.; Essigmann J. M., Effect of Sequence Context on O6-Methylguanine Repair and Replication in Vivo. Biochemistry 2001, 40 (49), 14968–14975. 11732917 10.1021/bi015578f

[38] Ken M. L., , RNA conformational propensities determine cellular activity. Nature 2023, 617 (7962), 835–841. 37198487 10.1038/s41586-023-06080-x PMC10429349

[39] Palmer A. G.; Massi F., Characterization of the Dynamics of Biomacromolecules Using Rotating-Frame Spin Relaxation NMR Spectroscopy. Chemical Reviews 2006, 106 (5), 1700–1719. 16683750 10.1021/cr0404287

[40] Rangadurai A., , Characterizing micro-to-millisecond chemical exchange in nucleic acids using off-resonance $R_{1\rho }$ relaxation dispersion. Prog Nucl Magn Reson Spectrosc 2019, 112–113, 55–102. 10.1016/j.pnmrs.2019.05.002 PMC6727989 31481159

[41] Alderson T. R.; Kay L. E., Unveiling invisible protein states with NMR spectroscopy. Curr Opin Struc Biol 2020, 60, 39–49. 10.1016/j.sbi.2019.10.008 31835059

[42] Freudenthal B. D., , Capturing snapshots of APE1 processing DNA damage. Nature Structural & Molecular Biology 2015, 22 (11), 924–931. 10.1038/nsmb.3105 PMC4654669 26458045

[43] Çağlayan M.; Wilson S. H., Pol μ dGTP mismatch insertion opposite T coupled with ligation reveals promutagenic DNA repair intermediate. Nature Communications 2018, 9 (1), 4213. 10.1038/s41467-018-06700-5 PMC6181931 30310068

[44] Tomasetti C.; Vogelstein B., Cancer etiology. Variation in cancer risk among tissues can be explained by the number of stem cell divisions. Science (New York, N.Y.) 2015, 347 (6217), 78–81. 25554788 10.1126/science.1260825 PMC4446723

[45] Tomasetti C., , Stem cell divisions, somatic mutations, cancer etiology, and cancer prevention. Science 2017, 355 (6331), 1330–1334. 28336671 10.1126/science.aaf9011 PMC5852673

[46] Schaaper R. M.; Dunn R. L., Spectra of spontaneous mutations in Escherichia coli strains defective in mismatch correction: the nature of in vivo DNA replication errors. Proc Natl Acad Sci U S A 1987, 84 (17), 6220–4. 3306672 10.1073/pnas.84.17.6220 PMC299042

[47] Kunkel T. A.; Bebenek K., Recent studies of the fidelity of DNA synthesis. Biochimica et Biophysica Acta (BBA) - Gene Structure and Expression 1988, 951 (1), 1–15. 2847793 10.1016/0167-4781(88)90020-6

[48] Halliday J. A.; Glickman B. W., Mechanisms of spontaneous mutation in DNA repair-proficient Escherichia coli. Mutation Research/Fundamental and Molecular Mechanisms of Mutagenesis 1991, 250 (1), 55–71. 1944363 10.1016/0027-5107(91)90162-h

[49] Kool E. T., , Mimicking the Structure and Function of DNA: Insights into DNA Stability and Replication. Angew Chem Int Ed 2000, 39 (6), 990–1009. 10.1002/(sici)1521-3773(20000317)39:6<990::aid-anie990>3.0.co;2-0 10760910

[50] Nakagawa H.; Fujita M., Whole genome sequencing analysis for cancer genomics and precision medicine. Cancer Science 2018, 109 (3), 513–522. 29345757 10.1111/cas.13505 PMC5834793

[51] Murphy F. V., , The role of modifications in codon discrimination by tRNALysUUU. Nature Structural & Molecular Biology 2004, 11 (12), 1186–1191. 10.1038/nsmb861 15558052

[52] Sochacka E., , 2-Thiouracil deprived of thiocarbonyl function preferentially base pairs with guanine rather than adenine in RNA and DNA duplexes. Nucleic Acids Res 2015, 43 (5), 2499–2512. 25690900 10.1093/nar/gkv109 PMC4357714

[53] Rozov A., , Novel base-pairing interactions at the tRNA wobble position crucial for accurate reading of the genetic code. Nature Communications 2016, 7 (1), 10457. 10.1038/ncomms10457 PMC4736104 26791911

[54] Sochacka E., , C5-substituents of uridines and 2-thiouridines present at the wobble position of tRNA determine the formation of their keto-enol or zwitterionic forms - a factor important for accuracy of reading of guanosine at the 3’-end of the mRNA codons. Nucleic Acids Res 2017, 45 (8), 4825–4836. 28088758 10.1093/nar/gkw1347 PMC5416851

[55] Westhof E., , Anionic G•U pairs in bacterial ribosomal rRNAs. RNA 2023, 29 (7), 1069–1076. 37068913 10.1261/rna.079583.123 PMC10275268

[56] Saon Md S., , Identification and characterization of shifted G•U wobble pairs resulting from alternative protonation of RNA. Nucleic Acids Res 2025, 53 (14). 10.1093/nar/gkaf575 PMC12282950 40694854

[57] Huff A. C.; Topal M. D., DNA damage at thymine N-3 abolishes base-pairing capacity during DNA synthesis. J Biol Chem 1987, 262 (26), 12843–50. 2442169

[58] Delaney J. C.; Essigmann J. M., Mutagenesis, genotoxicity, and repair of 1-methyladenine, 3-alkylcytosines, 1-methylguanine, and 3-methylthymine in *alkB Escherichia coli*. PNAS 2004, 101 (39), 14051–14056. 15381779 10.1073/pnas.0403489101 PMC521119

[59] Shirmanova M. V., , Intracellular pH imaging in cancer cells in vitro and tumors in vivo using the new genetically encoded sensor SypHer2. Biochimica et Biophysica Acta (BBA) - General Subjects 2015, 1850 (9), 1905–1911. 25964069 10.1016/j.bbagen.2015.05.001

[60] Liu Y., , Intracellular pH Regulates Cancer and Stem Cell Behaviors: A Protein Dynamics Perspective. Frontiers in Oncology 2020, *Volume* 10 – 2020. 10.3389/fonc.2020.01401 PMC7479815 32983969

[61] Sowers L. C., , Equilibrium between a wobble and ionized base pair formed between fluorouracil and guanine in DNA as studied by proton and fluorine NMR. J Biol Chem 1988, 263 (29), 14794–14801. 3170564

[62] Siegfried N. A., , Driving forces for nucleic acid pK(a) shifting in an A(+).C wobble: effects of helix position, temperature, and ionic strength. Biochemistry 2010, 49 (15), 3225–36. 20337429 10.1021/bi901920g

[63] Saenger W., Physical Properties of Nucleotides: Charge Densities, pK Values, Spectra, and Tautomerism. In Principles of Nucleic Acid Structure, Springer New York: New York, NY, 1984; pp 105–115.

[64] Kremer A. B., , Chemical consequences of the incorporation of 5-fluorouracil into DNA as studied by NMR. Biochemistry 1987, 26 (2), 391–397. 3828314 10.1021/bi00376a009

[65] Sowers L. C., , Structural and dynamic properties of a fluorouracil-adenine base pair in DNA studied by proton NMR. J Biol Chem 1987, 262 (32), 15436–42. 3680205

[66] Sowers L. C., , DNA base modification: Ionized base pairs and mutagenesis. Mutation Research/Fundamental and Molecular Mechanisms of Mutagenesis 1987, 177 (2), 201–218. 3561423 10.1016/0027-5107(87)90003-0

[67] Acharya P., , Measurement of Nucleobase pKa Values in Model Mononucleotides Shows RNA−RNA Duplexes To Be More Stable than DNA−DNA Duplexes. J Am Chem Soc 2004, 126 (9), 2862–2869. 14995203 10.1021/ja0386546

[68] Knobloch B., , Metal ion-binding properties of (N3)-deprotonated uridine, thymidine, and related pyrimidine nucleosides in aqueous solution. PNAS 2005, 102 (21), 7459–7464. 15897459 10.1073/pnas.0501446102 PMC1140430

[69] Chatterjee S., , The chemical nature of the 2’-substituent in the pentose-sugar dictates the pseudoaromatic character of the nucleobase (pKa) in DNA/RNA. Organic & Biomolecular Chemistry 2006, 4 (9), 1675–1686. 16633560 10.1039/b601460g

[70] Thaplyal P.; Bevilacqua P. C., Chapter Nine - Experimental Approaches for Measuring pKa’s in RNA and DNA. In Methods in Enzymology, Burke-Aguero D. H., Ed. Academic Press: 2014; Vol. 549, pp 189–219. 25432750 10.1016/B978-0-12-801122-5.00009-X PMC5597436

[71] González-Olvera J. C., , Determination of pKa values for deprotonable nucleobases in short model oligonucleotides. Biophysical Chemistry 2015, 206, 58–65. 26188860 10.1016/j.bpc.2015.07.001

[72] Scott L. G.; Hennig M., Chapter Three - 19F-Site-Specific-Labeled Nucleotides for Nucleic Acid Structural Analysis by NMR. In Methods in Enzymology, Kelman Z., Ed. Academic Press: 2016; Vol. 566, pp 59–87. 26791976 10.1016/bs.mie.2015.05.015

[73] Gakh Y. G., , Fluorine as an NMR probe for structural studies of chemical and biological systems. Magn Reson Chem 2000, 38 (7), 551–558.

[74] Gronenborn A. M., Small, but powerful and attractive: ^19^F in biomolecular NMR. Structure 2022, 30 (1), 6–14. 34995480 10.1016/j.str.2021.09.009 PMC8797020

[75] Wijmenga S. S., , Analysis of 1H chemical shifts in DNA: Assessment of the reliability of 1H chemical shift calculations for use in structure refinement. J Biomol NMR 1997, 10 (4), 337–350. 20859781 10.1023/A:1018348123074

[76] Dejaegere A., , An Empirical Analysis of Proton Chemical Shifts in Nucleic Acids. In Modeling NMR Chemical Shifts, American Chemical Society: 1999; Vol. 732, pp 194–206.

[77] Case D. A., Interpretation of chemical shifts and coupling constants in macromolecules. Curr Opin Struc Biol 2000, 10 (2), 197–203. 10.1016/s0959-440x(00)00068-3 10753812

[78] Puffer B., , 5-Fluoro pyrimidines: labels to probe DNA and RNA secondary structures by 1D ^19^F NMR spectroscopy. Nucleic Acids Res 2009, 37 (22), 7728–7740. 19843610 10.1093/nar/gkp862 PMC2794194

[79] Parker J. B.; Stivers J. T., Dynamics of Uracil and 5-Fluorouracil in DNA. Biochemistry 2011, 50 (5), 612–617. 21190322 10.1021/bi101536k PMC3079343

[80] Becette O. B., , Solution NMR readily reveals distinct structural folds and interactions in doubly ^13^C- and ^19^F-labeled RNAs. Science Advances 2020, 6 (41), eabc6572. 33028531 10.1126/sciadv.abc6572 PMC7541061

[81] Lee Y., , Kinetic Dissection of Proton-Coupled Conformational Transitions in Nucleic Acids by Integrating pH-Dependent NMR and Chemical Modifications. J Am Chem Soc 2025, 147 (23), 19643–19666. 40423646 10.1021/jacs.5c01663 PMC13102259

[82] Rangadurai A., , Probing conformational transitions towards mutagenic Watson–Crick-like G•T mismatches using off-resonance sugar carbon $R_{1\rho }$ relaxation dispersion. J Biomol NMR 2020, 74 (8), 457–471. 32789613 10.1007/s10858-020-00337-7 PMC7508749

[83] Johnson S. J.; Beese L. S., Structures of Mismatch Replication Errors Observed in a DNA Polymerase. Cell 2004, 116 (6), 803–816. 15035983 10.1016/s0092-8674(04)00252-1

[84] Lee Y., , Insights into the A-C Mismatch Conformational Ensemble in Duplex DNA and its Role in Genetic Processes through a Structure-based Review. J Mol Biol 2024, 436 (18), 168710. 39009073 10.1016/j.jmb.2024.168710 PMC12034297

[85] Kimsey I. J., , Visualizing transient Watson-Crick-like mispairs in DNA and RNA duplexes. Nature 2015, 519 (7543), 315–20. 25762137 10.1038/nature14227 PMC4547696

[86] Robinson H., , 2’-Deoxyisoguanosine Adopts More than One Tautomer To Form Base Pairs with Thymidine Observed by High-Resolution Crystal Structure Analysis. Biochemistry 1998, 37 (31), 10897–10905. 9692982 10.1021/bi980818l

[87] Geng A., , Insight into the Conformational Ensembles Formed by U–U and T–T Mismatches in RNA and DNA Duplexes From a Structure-based Survey, NMR, and Molecular Dynamics Simulations. J Mol Biol 2025, 437 (17), 169197. 40345379 10.1016/j.jmb.2025.169197

[88] Szymanski E. S., , Direct NMR Evidence that Transient Tautomeric and Anionic States in dG•dT Form Watson-Crick-like Base Pairs. J Am Chem Soc 2017, 139 (12), 4326–4329. 28290687 10.1021/jacs.7b01156 PMC5581979

[89] Kobyłecka M., , Valence Anion of Thymine in the DNA π-Stack. J Am Chem Soc 2008, 130 (46), 15683–15687. 18954049 10.1021/ja806251h

[90] Schottel B. L., , Anion-π interactions. Chemical Society Reviews 2008, 37 (1), 68–83. 18197334 10.1039/b614208g

[91] Wang D.-X.; Wang M.-X., Anion−π Interactions: Generality, Binding Strength, and Structure. J Am Chem Soc 2013, 135 (2), 892–897. 23244296 10.1021/ja310834w

[92] Lucas X., , A thorough anion–π interaction study in biomolecules: on the importance of cooperativity effects. Chemical Science 2016, 7 (2), 1038–1050. 29899893 10.1039/c5sc01386k PMC5967298

[93] Chawla M., , Occurrence and stability of anion–π interactions between phosphate and nucleobases in functional RNA molecules. Nucleic Acids Res 2022, 50 (20), 11455–11469. 36416268 10.1093/nar/gkac1081 PMC9723503

[94] Allawi H. T.; SantaLucia J., Thermodynamics and NMR of Internal G•T Mismatches in DNA. Biochemistry 1997, 36 (34), 10581–10594. 9265640 10.1021/bi962590c

[95] Watkins N. E. Jr; SantaLucia J. Jr, Nearest-neighbor thermodynamics of deoxyinosine pairs in DNA duplexes. Nucleic Acids Res 2005, 33 (19), 6258–6267. 16264087 10.1093/nar/gki918 PMC1277807

[96] Acharya S., , Significant pKa Perturbation of Nucleobases Is an Intrinsic Property of the Sequence Context in DNA and RNA. J Am Chem Soc 2004, 126 (28), 8674–8681. 15250719 10.1021/ja048484c

[97] Yang S., , Measuring similarity between dynamic ensembles of biomolecules. Nature Methods 2014, 11 (5), 552–554. 24705474 10.1038/nmeth.2921 PMC4041546

[98] Talhaoui I., , Aberrant repair initiated by mismatch-specific thymine-DNA glycosylases provides a mechanism for the mutational bias observed in CpG islands. Nucleic Acids Res 2014, 42 (10), 6300–6313. 24692658 10.1093/nar/gku246 PMC4041421

[99] Fu D., , Balancing repair and tolerance of DNA damage caused by alkylating agents. Nature Reviews Cancer 2012, 12 (2), 104–120. 22237395 10.1038/nrc3185 PMC3586545

[100] Kucab J. E., , A Compendium of Mutational Signatures of Environmental Agents. Cell 2019, 177 (4), 821–836.e16. 30982602 10.1016/j.cell.2019.03.001 PMC6506336

[101] Armijo A. L., , Molecular origins of mutational spectra produced by the environmental carcinogen N-nitrosodimethylamine and SN1 chemotherapeutic agents. NAR Cancer 2023, 5 (2). 10.1093/narcan/zcad015 PMC10041537 36992846

[102] Patel D. J., , Structural studies of the O6meG.cntdot.T interaction in the d(C-G-T-G-A-A-T-T-C-O6meG-C-G) duplex. Biochemistry 1986, 25 (5), 1036–1042. 3964659 10.1021/bi00353a013

[103] Leonard G. A., , High-resolution structure of a mutagenic lesion in DNA. *PNAS* 1990, 87 (24), 9573–9576. 2263612 10.1073/pnas.87.24.9573 PMC55214

[104] Esteller M., , Inactivation of the DNA repair gene O6-methylguanine-DNA methyltransferase by promoter hypermethylation is associated with G to A mutations in K-ras in colorectal tumorigenesis. Cancer Res 2000, 60 (9), 2368–71. 10811111

[105] Dolan M. E., , Sequence specificity of guanine alkylation and repair. Carcinogenesis 1988, 9 (11), 2139–2143. 3180351 10.1093/carcin/9.11.2139

[106] Alexandrov L. B., , Clock-like mutational processes in human somatic cells. Nature Genetics 2015, 47 (12), 1402–1407. 26551669 10.1038/ng.3441 PMC4783858

[107] Nik-Zainal S., , Landscape of somatic mutations in 560 breast cancer whole-genome sequences. Nature 2016, 534 (7605), 47–54. 27135926 10.1038/nature17676 PMC4910866

[108] Feng S., , Conservation and divergence of methylation patterning in plants and animals. PNAS 2010, 107 (19), 8689–8694. 20395551 10.1073/pnas.1002720107 PMC2889301

[109] Choi J. K., Contrasting chromatin organization of CpG islands and exons in the human genome. Genome Biology 2010, 11 (7), R70. 20602769 10.1186/gb-2010-11-7-r70 PMC2926781

[110] Maunakea A. K., , Intragenic DNA methylation modulates alternative splicing by recruiting MeCP2 to promote exon recognition. Cell Res 2013, 23 (11), 1256–1269. 23938295 10.1038/cr.2013.110 PMC3817542

[111] Almatarneh M. H., , Computational Study of the Deamination Reaction of Cytosine with H2O and OH. The Journal of Physical Chemistry A 2006, 110 (26), 8227–8234. 16805511 10.1021/jp062300u

[112] Cannistraro V. J.; Taylor J.-S., Acceleration of 5-Methylcytosine Deamination in Cyclobutane Dimers by G and Its Implications for UV-Induced C-to-T Mutation Hotspots. J Mol Biol 2009, 392 (5), 1145–1157. 19631218 10.1016/j.jmb.2009.07.048 PMC3026386

[113] Carpenter M., , Sequence-dependent enhancement of hydrolytic deamination of cytosines in DNA by the restriction enzyme PspGI. Nucleic Acids Res 2006, 34 (13), 3762–3770. 16893959 10.1093/nar/gkl545 PMC1557792

[114] Sibghat U., , Base Analog and Neighboring Base Effects on Substrate Specificity of Recombinant Human G:T Mismatch-Specific Thymine DNA−Glycosylase. Biochemistry 1996, 35 (39), 12926–12932. 8841138 10.1021/bi961022u

[115] Davies H., , HRDetect is a predictor of BRCA1 and BRCA2 deficiency based on mutational signatures. Nat Med 2017, 23 (4), 517–525. 28288110 10.1038/nm.4292 PMC5833945

[116] Chen D., , BRCA1 deficiency specific base substitution mutagenesis is dependent on translesion synthesis and regulated by 53BP1. Nature Communications 2022, 13 (1), 226. 10.1038/s41467-021-27872-7 PMC8752635 35017534

[117] Zámborszky J., , Loss of BRCA1 or BRCA2 markedly increases the rate of base substitution mutagenesis and has distinct effects on genomic deletions. Oncogene 2017, 36 (6), 746–755. 27452521 10.1038/onc.2016.243 PMC5096687

[118] Al-Moghrabi N., , Methylation of BRCA1 and MGMT genes in white blood cells are transmitted from mothers to daughters. Clinical Epigenetics 2018, 10 (1), 99. 30049288 10.1186/s13148-018-0529-5 PMC6062990

[119] Hodel K. P., , POLE Mutation Spectra Are Shaped by the Mutant Allele Identity, Its Abundance, and Mismatch Repair Status. Molecular Cell 2020, 78 (6), 1166–1177.e6. 32497495 10.1016/j.molcel.2020.05.012 PMC8177757

[120] Carmical J. R., , Mutagenic potential of adenine N6 adducts of monoepoxide and diolepoxide derivatives of butadiene. Environmental and Molecular Mutagenesis 2000, 35 (1), 48–56. 10692227

[121] Khalili H., , Mutagenicity of benzo[a]pyrene–deoxyadenosine adducts in a sequence context derived from the p53 gene. Mutation Research/Genetic Toxicology and Environmental Mutagenesis 2000, 465 (1), 39–44. 10.1016/s1383-5718(99)00203-x 10708967

[122] Nair D. T., , Hoogsteen base pair formation promotes synthesis opposite the 1,N6-ethenodeoxyadenosine lesion by human DNA polymerase ι. Nature Structural & Molecular Biology 2006, 13 (7), 619–625. 10.1038/nsmb1118 16819516

[123] Conde J., , Genetic Control of Replication through N1-methyladenine in Human Cells. J Biol Chem 2015, 290 (50), 29794–29800. 26491020 10.1074/jbc.M115.693010 PMC4705973

[124] Patra A., , Structural and Kinetic Analysis of Miscoding Opposite the DNA Adduct 1,N^6^-Ethenodeoxyadenosine by Human Translesion DNA Polymerase η. J Biol Chem 2016, 291 (27), 14134–14145. 27226627 10.1074/jbc.M116.732487 PMC4933172

[125] Nik-Zainal S., , The genome as a record of environmental exposure. Mutagenesis 2015, 30 (6), 763–770. 26443852 10.1093/mutage/gev073 PMC4637815

[126] Ma B., , Recent Studies on DNA Adducts Resulting from Human Exposure to Tobacco Smoke. Toxics 2019, 7 (1), 16. 30893918 10.3390/toxics7010016 PMC6468371

[127] Lawson A. R. J., , Extensive heterogeneity in somatic mutation and selection in the human bladder. Science 2020, 370 (6512), 75–82. 33004514 10.1126/science.aba8347

[128] Mingard C., , Dissection of Cancer Mutational Signatures with Individual Components of Cigarette Smoking. Chemical Research in Toxicology 2023, 36 (4), 714–723. 36976926 10.1021/acs.chemrestox.3c00021 PMC10114081

[129] Wei S. J., , Dose-dependent differences in the profile of mutations induced by an ultimate carcinogen from benzo[a]pyrene. PNAS 1991, 88 (24), 11227–11230. 1763036 10.1073/pnas.88.24.11227 PMC53107

[130] Wei S.-J. C., , Dose-dependent Differences in the Profile of Mutations Induced by (+)-7R,8S-Dihydroxy-9S,10R-epoxy-7,8,9,10-tetrahydrobenzo(a)pyrene in the Coding Region of the Hypoxanthine (Guanine) Phosphoribosyltransferase Gene in Chinese Hamster V-79 Cells1. Cancer Research 1993, 53 (14), 3294–3301. 8324741

[131] Delaney J. C., , AlkB reverses etheno DNA lesions caused by lipid oxidation in vitro and in vivo. Nature Structural & Molecular Biology 2005, 12 (10), 855–860. 10.1038/nsmb996 16200073

[132] Dylewska M., , 1,N6-α-hydroxypropanoadenine, the acrolein adduct to adenine, is a substrate for AlkB dioxygenase. Biochemical Journal 2017, 474 (11), 1837–1852. 28408432 10.1042/BCJ20161008

[133] De los Santos C., , NMR studies of the exocyclic 1,N6-ethenodeoxyadenosine adduct (.epsilon.dA) opposite deoxyguanosine in a DNA duplex. .epsilon.dA(syn).cntdot.dG(anti) pairing at the lesion site. Biochemistry 1991, 30 (7), 1828–1835. 1993197 10.1021/bi00221a015

[134] Volk D. E., , NMR Evidence for Syn-Anti Interconversion of a Trans Opened (10R)-dA Adduct of Benzo[a]pyrene (7S,8R)-Diol (9R,10S)-Epoxide in a DNA Duplex. Biochemistry 2000, 39 (46), 14040–14053. 11087351 10.1021/bi001669l

[135] Pradhan P., , Solution Structure of a Trans-Opened (10S)-dA Adduct of (+)-(7S,8R,9S,10R)-7,8-Dihydroxy-9,10-epoxy-7,8,9,10-tetrahydrobenzo[a]pyrene in a Fully Complementary DNA Duplex: Evidence for a Major Syn Conformation. Biochemistry 2001, 40 (20), 5870–5881. 11352722 10.1021/bi002896q

[136] Ling H., , Crystal structure of a benzo[a]pyrene diol epoxide adduct in a ternary complex with a DNA polymerase. PNAS 2004, 101 (8), 2265–2269. 14982998 10.1073/pnas.0308332100 PMC356939

[137] Yang H., , Effect of 1-methyladenine on double-helical DNA structures. FEBS Letters 2008, 582 (11), 1629–1633. 18435925 10.1016/j.febslet.2008.04.013

[138] Sathyamoorthy B., , Insights into Watson–Crick/Hoogsteen breathing dynamics and damage repair from the solution structure and dynamic ensemble of DNA duplexes containing m1A. Nucleic Acids Res 2017, 45 (9), 5586–5601. 28369571 10.1093/nar/gkx186 PMC5435913

[139] Donny-Clark K.; Broyde S., Influence of local sequence context on damaged base conformation in human DNA polymerase ι: molecular dynamics studies of nucleotide incorporation opposite a benzo[a]pyrene-derived adenine lesion. Nucleic Acids Res 2009, 37 (21), 7095–7109. 19767609 10.1093/nar/gkp745 PMC2790882

[140] Gowda A. S. P., , Mutagenic Replication of N2-Deoxyguanosine Benzo[a]pyrene Adducts by Escherichia coli DNA Polymerase I and Sulfolobus solfataricus DNA Polymerase IV. Chemical Research in Toxicology 2017, 30 (5), 1168–1176. 28402640 10.1021/acs.chemrestox.6b00466 PMC5673090

[141] Hoang M. L., , Mutational Signature of Aristolochic Acid Exposure as Revealed by Whole-Exome Sequencing. Science Translational Medicine 2013, 5 (197), 197ra102–197ra102. 10.1126/scitranslmed.3006200 PMC3973132 23926200

[142] Poon S. L., , Genome-wide mutational signatures of aristolochic acid and its application as a screening tool. Sci Transl Med 2013, 5 (197), 197ra101. 10.1126/scitranslmed.3006086 23926199

[143] Senkin S., , Geographic variation of mutagenic exposures in kidney cancer genomes. Nature 2024, 629 (8013), 910–918. 38693263 10.1038/s41586-024-07368-2 PMC11111402

[144] Kathuria P., , Conformational Preferences of DNA following Damage by Aristolochic Acids: Structural and Energetic Insights into the Different Mutagenic Potential of the ALI and ALII-N6-dA Adducts. Biochemistry 2015, 54 (15), 2414–2428. 25761009 10.1021/bi501484m

[145] Kathuria P., , Adenine versus guanine DNA adducts of aristolochic acids: role of the carcinogen–purine linkage in the differential global genomic repair propensity. Nucleic Acids Res 2015, 43 (15), 7388–7397. 26175048 10.1093/nar/gkv701 PMC4551933

[146] Lu H., , Aristolochic acid mutational signature defines the low-risk subtype in upper tract urothelial carcinoma. Theranostics 2020, 10 (10), 4323–4333. 32292497 10.7150/thno.43251 PMC7150494

[147] Inskeep P. B., , Covalent binding of 1,2-dihaloalkanes to DNA and stability of the major DNA adduct, S-[2-(N7-guanyl)ethyl]glutathione. Cancer Res 1986, 46 (6), 2839–44. 2870801

[148] Humphreys W. G., , Comparison of the DNA-alkylating properties and mutagenic responses of a series of S-(2-haloethyl)-substituted cysteine and glutathione derivatives. Biochemistry 1990, 29 (45), 10342–50. 2261477 10.1021/bi00497a008

[149] Mitchell E., , The long-term effects of chemotherapy on normal blood cells. Nature Genetics 2025, 57 (7), 1684–1694. 40596443 10.1038/s41588-025-02234-x PMC12283364

[150] Akiba N., , Influence of GSH S-transferase on the mutagenicity induced by dichloromethane and 1,2-dichloropropane. Mutagenesis 2017, 32 (4), 455–462. 28521016 10.1093/mutage/gex014

[151] Toyooka T., , 1,2-Dichloropropane generates phosphorylated histone H2AX via cytochrome P450 2E1-mediated metabolism. Toxicology Letters 2017, 272, 60–67. 28300663 10.1016/j.toxlet.2017.03.009

[152] Zong C., , Exposure to 1,2-Dichloropropane Upregulates the Expression of Activation-Induced Cytidine Deaminase (AID) in Human Cholangiocytes Co-Cultured With Macrophages. Toxicological Sciences 2018, 168 (1), 137–148. 10.1093/toxsci/kfy280 30452740

[153] Buranarom A., , Dichloromethane increases mutagenic DNA damage and transformation ability in cholangiocytes and enhances metastatic potential in cholangiocarcinoma cell lines. Chemico-Biological Interactions 2021, 346, 109580. 34280354 10.1016/j.cbi.2021.109580

[154] Kim D. H., , Characterization of S-[2-(N1-adenyl)ethyl]glutathione as an adduct formed in RNA and DNA from 1,2-dibromoethane. Chemical Research in Toxicology 1990, 3 (6), 587–594. 1715767 10.1021/tx00018a015

[155] Cho S.-H.; Guengerich F. P., In Vivo Roles of Conjugation with Glutathione and O6-Alkylguanine DNA-Alkyltransferase in the Mutagenicity of the Bis-Electrophiles 1,2-Dibromoethane and 1,2,3,4-Diepoxybutane in Mice. Chemical Research in Toxicology 2013, 26 (11), 1765–1774. 24191644 10.1021/tx4003534 PMC3889014

[156] Szekely O., , NMR measurements of transient low-populated tautomeric and anionic Watson–Crick-like G•T/U in RNA:DNA hybrids: implications for the fidelity of transcription and CRISPR/Cas9 gene editing. Nucleic Acids Res 2024, 52 (5), 2672–2685. 38281263 10.1093/nar/gkae027 PMC10954477

[157] Rozov A., , The ribosome prohibits the G•U wobble geometry at the first position of the codon-anticodon helix. Nucleic Acids Res 2016, 44 (13), 6434–41. 27174928 10.1093/nar/gkw431 PMC5291260

[158] Pacesa M., , Structural basis for Cas9 off-target activity. Cell 2022, 185 (22), 4067–4081.e21. 36306733 10.1016/j.cell.2022.09.026 PMC10103147

[159] Wilcox J. L.; Bevilacqua P. C., pKa Shifting in Double-Stranded RNA Is Highly Dependent upon Nearest Neighbors and Bulge Positioning. Biochemistry 2013, 52 (42), 7470–7476. 24099082 10.1021/bi400768q

[160] Delaglio F., , NMRPipe: A multidimensional spectral processing system based on UNIX pipes. J Biomol NMR 1995, 6 (3), 277–293. 8520220 10.1007/BF00197809

[161] Lee W., , NMRFAM-SPARKY: enhanced software for biomolecular NMR spectroscopy. Bioinformatics 2015, 31 (8), 1325–7. 25505092 10.1093/bioinformatics/btu830 PMC4393527

[162] Korzhnev D. M., , Off-Resonance $R_{1\rho }$ NMR Studies of Exchange Dynamics in Proteins with Low Spin-Lock Fields: An Application to a Fyn SH3 Domain. J Am Chem Soc 2005, 127 (2), 713–721. 15643897 10.1021/ja0446855

[163] Nikolova E. N., , Probing transient Hoogsteen hydrogen bonds in canonical duplex DNA using NMR relaxation dispersion and single-atom substitution. J Am Chem Soc 2012, 134 (8), 3667–70. 22309937 10.1021/ja2117816 PMC3791138

[164] McConnell H. M., Reaction Rates by Nuclear Magnetic Resonance. The Journal of Chemical Physics 1958, 28 (3), 430–431.

[165] Bothe J. R., , Evaluating the uncertainty in exchange parameters determined from off-resonance $R_{1\rho }$ relaxation dispersion for systems in fast exchange. J Magn Reson 2014, 244, 18–29. 24819426 10.1016/j.jmr.2014.04.010 PMC4222517

[166] MenØndez M. L., , The Jensen-Shannon divergence. Journal of the Franklin Institute 1997, 334 (2), 307–318.

[167] David F. KLDIV. https://www.mathworks.com/matlabcentral/fileexchange/13089-kldiv (accessed 05/17/2024).

